# The Characterization of the Key Aroma Compounds in Non-Smoked Bacon by Instrumental and Sensory Methods

**DOI:** 10.3390/foods13081260

**Published:** 2024-04-19

**Authors:** Han Wu, Zhifei He, Li Yang, Hongjun Li

**Affiliations:** 1 College of Food Science, Chongqing Engineering Research Center of Regional Food, Southwest University, No.2 Tiansheng Road, Beibei District, Chongqing 400715, China; 15137178310@163.com (H.W.); 18102309010@163.com (Z.H.); 17823232400@163.com (L.Y.); 2 Chongqing Key Laboratory of Speciality Food Co-Built by Sichuan and Chongqing, Chongqing 400715, China

**Keywords:** non-smoked bacon, key aroma compound, GC–O, GC × GC–TOFMS, GC–IMS

## Abstract

The aroma profiles in non-smoked bacon were investigated via GC–O–MS, GC × GC–TOFMS, and GC–IMS. GC-O-MS is advantageous for detecting aldehydes. GC × GC-TOFMS is more sensitive to hydrocarbons and alcohols, while GC-IMS detects a balanced range of categories. Only 9 of the 239 detected volatiles were identifiable by all three methods. Therefore, the combination of all three methods proved to be the most effective way to comprehensively analyze the aroma profiles of bacon. Recombination and omission tests were performed using aroma compounds with a flavor dilution (FD) factor greater than 27; five volatiles were identified as key aroma compounds in non-smoked bacon, including hexanal, (E,E)-2,4-decadienal, 1-octen-3-ol, dihydro-5-pentyl-2(3H)-furanone, and 3-methyl-butanoic acid. Among these, hexanal and 1-octen-3-ol exhibited relatively high FD factors and odor activity values (OAVs), so they were confirmed as the primary contributors. Meanwhile, seven volatiles contributed to the unique aroma of non-smoked bacon in different regions. The difference in the aroma of bacon in different regions is mainly due to the content of various volatiles rather than the type. A comprehensive analysis of the aroma in non-smoked bacon can reveal theoretical information for improving the process and quality control of the product.

## 1. Introduction

Traditional Chinese bacon is a widely consumed dry-cured meat product in southern China. It is typically prepared using belly meat, which undergoes salting, smoking or non-smoking, and drying [1]. Locals in China have shown a preference for a variety of traditional bacon styles, such as those originating from Sichuan, Yunnan, Jiangxi, Chongqing, and Guizhou [2]. The aroma of non-smoked bacon is a vital factor in the evaluation of the bacon quality and affects consumer satisfaction. Volatile compounds in dry-cured meat primarily originate from protein hydrolysis, lipid degradation, oxidation, and Strecker degradation [3]. Due to the different conditions of the manufacturing process and the breeds of pig, non-smoked bacons from different regions present distinct sensory characteristics [4]. Yunnan, Chongqing, and Sichuan province were the main production areas of bacon. Analyzing the volatile compounds present in bacon from these regions aids in comprehensively understanding the key aroma compounds found in non-smoked bacon.

Research on volatile compounds’ profiles of bacon was mainly focused on smoked bacon. Using gas chromatography–olfactometry alongside aroma extract dilution analysis (GC–O/AEDA), previous research has determined 39 important aroma compounds in traditional smoked pork leg from Hunan. The key aromas in the smoked pork leg were proven to be (E)-2-nonenal, 2-methoxy-4-vinylphenol, guaiacol, 3-ethylphenol, 2,6-dimethylphenol, 2-acetyl-1-pyrroline, and methional by the recombination and omission test [5]. In smoked cooked loin, researchers identified 27 volatile molecules with significant odor activity [6]. A prior study examined the aroma compounds of Chinese smoked bacon via the nitrogen purge-and-steam distillation (NPSD) technique [7]. The study’s findings suggest that the predominant volatile compounds in smoked bacon are phenolic derivatives. For the aroma of non-smoked bacon, prior studies have found that lipolysis and lipid oxidation are responsible for 47.37–50.85% of the typical aroma. Additionally, 1-octen-3-ol and hexanal have been identified as the primary aroma compounds [7]. Headspace chromatography–ion mobility spectrometry (HS-GC-IMS) was utilized to recognize volatile compounds in non-smoked bacon [1]. The study’s results demonstrate that elevating the levels of nonanal, octanal, heptanal, 3-methylbutanal, n-hexyl acetate, and n-propyl acetate may enhance the aroma attributes of non-smoked bacon. Although some volatiles were considered crucial aroma compounds, the results were all relative and lacked conclusions combined with sensory evaluation. Rare studies identified key aroma compounds in non-smoked bacon via AEDA and recombination and omission experiments.

The aroma of a food commodity is a crucial sensory feature, and methods of instrumental analysis are continually improved to identify volatiles with greater comprehensiveness and precision. GC–IMS is a powerful analytical technique with a low detection limit and less pretreatment, and it is commonly used in the aroma analysis of food under normal atmospheric pressure. Previous research investigated the composition of volatile compounds, food classification and adulteration, and off-flavor detection via GC–IMS, including coffee, table grapes, and water-boiled salted duck [8,9,10,11]. Two-dimensional gas chromatography combined with time-of-flight mass spectrometry (GC × GC-TOFMS) offers high resolution and remarkable sensitivity by linking two chromatographic columns with varying characteristics through a modulator. This method can separate trace volatiles from complex mixtures, especially for overlapping peaks due to a similar polarity or boiling point of volatile compounds. GC × GC-MS analysis detected the existence of 50 volatile compounds in Laoshan green teas [12]. Certain volatile compounds, namely (Z)-carvyl acetate, carvone, δ-valerolactone, and α-damascenone, were exclusively identified through GC × GC–MS analysis and not observed using GC–MS. Considering the intricate nature of volatile compounds within food, utilizing a range of equipment, including GC–IMS and GC × GC–TOFMS, is now a frequently observed method to comprehensively analyze the characteristics of food aroma. The aroma compounds in chestnut-like aroma green tea were analyzed using a combination of GC–IMS, GC–E–nose, and GC × GC–TOFMS [13]. A total of 51 volatile biomarkers were identified out of the 211 volatiles detected. Additionally, the aromas in cold and hot break tomato pastes were characterized through the use of GC–O–MS, GC × GC–O–TOFMS, and GC–IMS [14]. This method has a broad applicability in a variety of food matrices to analyze aroma compounds and food classification, including moso bamboo leaf, Chinese dry-cured hams, and watermelon juice [3,14,15,16].

Consequently, this study aimed to (1) conduct a comprehensive analysis of the aroma of non-smoked bacon using GC-IMS, GC-O-MS, and GC × GC-TOFMS, and compare the characteristics of the three methods and (2) determine the key aroma compounds of non-smoked bacon from different regions through recombination and omission experiments and compare their similarities and differences. The study provided in-depth assessments of the aroma compounds in bacon from different regions using molecular sensory methods to reveal the key aroma compounds in non-smoked bacon.

## 2. Materials and Methods

### 2.1. Samples

Three non-smoked bacons produced in Chongqing (ZZ), Yunnan (XW), and Sichuan (MN) were purchased from the local supermarket (Chongqing, China), and three batches (replicates) were collected to make the study robust. The procedure for preparing non-smoked bacon involved cutting the pork belly to dimensions of 20 cm × 5 cm × 4 cm (length × width × thickness). For ZZ bacon, the belly was dry-cured with 4% salt (*m*/*m*) at °C for 3 days and dried by hot air at 55 °C for 24 h. For XW bacon, the belly was dry-cured with 10% salt (*m*/*m*) at 4 °C for 2 days and dried by hot air at 60 °C for 12 h. For MN bacon, the belly was dry-cured with 10% salt (*m*/*m*) at 4 °C for 3 days and dried by hot air at 40 °C for 48 h. Each sample was vacuum-sealed, and testing concluded within a month of sample production.

### 2.2. HS–SPME–GC–MS–O Analysis

#### 2.2.1. HS–SPME–GC–MS Analysis

An approach that has been modified was used to assess the aroma in traditional Chinese bacon [17]. Six grams of skinless, non-smoked bacon was finely minced and transferred into a 40 mL headspace vial. An internal standard of 2-methyl-3-heptanone (1 μL, 1.632 g/L) was subsequently added, and the vial was immersed in a water bath at 50 °C for 30 min. Solid-phase microextraction (SPME) fiber (50/30 μm, DVB/CAR/PDMS, Supelco, Bellefonte, PA, USA) was used to extract volatiles from samples for 40 min. The volatiles were then desorbed from the fiber by inserting them into the GC–MS (GCMS-QP2020 NX, Shimadzu, Kyoto, Japan) injector for 5 min at 250 °C. Pure helium served as a carrier gas to separate volatiles in an SH-Stabilwax-MS and SH-Rxi-5Sil MS capillary column (30 m × 0.25 mm, 0.25 μm, Shimadzu, Kyoto, Japan) with the splitless mode. The flow rate was set to 1.0 mL/min, and the oven temperature procedure was carried out as follows: the starting column temperature was held at 40 °C for 3 min, then raised to 55 °C at 2 °C/min and held for 3 min, then raised to 70 °C at 2 °C/min, and then raised to 130 °C at 5 °C/min. Ultimately, the temperature was further increased to 230 °C/min at a rate of 10 °C/min and held for a period of 2 min. Electron impact mass spectra were generated using an ion source (70 eV) at a temperature of 230 °C, with a complete scan mode range of 30 *m*/*z* to 500 *m*/*z*.

#### 2.2.2. Sensory Analysis by GC–O

An olfactometer coupled with GC–MS was used to analyze the key aroma compounds in bacon. After chromatographic column separation, the volatiles were split into an MS detector and an olfactometer. This research altered the split ratio of the GC interface, which had been initially set as 1, 1:3, 1:9, 1:27, 1:81, and 1:243, in order to carry out AEDA. This method has been proven to be an effective way to perform AEDA and analyze key aroma compounds in the sample [18]. The general aroma was a continuous gradient dilution until the aroma compounds could not be detected by the human nose. The FD factor was utilized to screen the key aroma compounds, with higher FD factors indicating a greater compound contribution.

#### 2.2.3. Identification and Quantitation Analysis of Volatile Compounds

The identification of the volatiles was performed via the mass spectrometry library (MS), odor qualities (O), retention indices (RI), and compared with authentic flavor standards (S). The volatile compounds were identified according to the NIST 17 database and confirmed by RI. The RI was determined following the same chromatographic conditions with the standard C6-C30 n-alkane series. The formula used to calculate the RI was as follows: RI = 100n + 100(t_x_ − t_n_)/(t_n+1_ − t_n_), where t_x_, t_n_, and t_n+1_ represented the retention times of compound x, alkane n, and alkane n + 1, respectively (t_n_ < t_x_ < t_n+1_). Some odorants can be validated by comparing their odor characteristics and retention times to those of authentic aroma standards under identical chromatographic conditions. The aroma compounds in non-smoked bacon were semi-quantitated via a mass concentration of an internal standard. The concentration of flavor compounds was determined by computing the peak area ratio of the internal standard and the target substances. To further quantify the key aroma compounds in bacon, the volatile compounds with an FD factor ≥27 were screened to construct the calibration curves. The process for establishing the calibration curves was as follows: mixing different concentrations of authentic aroma compounds with an artificial odorless matrix, which were then analyzed through gas chromatography–selected ion monitoring (GC–SIM). The calibration curves represent the concentration ratio of volatile compounds to the internal standard and the peak area ratio of standard peak area to the internal standard as x and y, respectively. The preparation of the artificial odorless matrix followed earlier research [19]. Briefly, the mixture of diethyl ether and pentane (2:1, *v*/*v*) was added to the minced bacon samples and agitated for 8 h. After that, the samples were freeze-dried for 48 h at −60 °C in a freeze dryer. The operation was repeated at least three times until no volatiles were detected by GC–MS–O. The internal standard’s recovery yield (*R*) was calculated to ensure accurate results. *R* is the ratio of the peak area of the internal standard added to the sample (Padded) to the peak area of the single internal standard (Preal). The recovery yield for 2-methyl-3-heptanone was 96.22%.

The odor activity value (OAV) can assess the impact of volatile compounds on the overall aroma. It is calculated as the concentration ratio in the samples to those in the water’s odor threshold.

### 2.3. GC–IMS Analysis

The aroma profiles of non-smoked bacon were analyzed via a GC–IMS instrument (FlavourSpec, G.A.S., Dortmund, Germany). Bacon samples were minced and placed in a 20 mL vial designed for headspace analysis. Then, they were incubated while being shaken and heated at 60 °C for 15 min (oscillation speed: 500 r/min). Following this, 500 µL of headspace was sampled and automatically injected into the injector via a syringe that had been heated to 85 °C. The volatiles were separated in a capillary column (MXT-5, 30 m × 0.53 mm, 1 µm) with nitrogen (99.99%). The flow was programmed according to the following parameters: initially 2.0 mL/min, 2–10 mL/min (at 2–5 min), 10–15 mL/min (at 5–15 min), 15–50 mL/min (at 15–20 min), and then 50–100 mL/min (at 20–30 min). The ions were transferred to the drift tube at a constant temperature of 45 °C, with a drift gas flow rate of 150 mL/min. Three parallel samples were analyzed for each bacon.

### 2.4. GC × GC–TOFMS Analysis

The analysis was carried out based on gas chromatography (Agilent 7890A, Agilent Technologies, Santa Clara, CA, USA) equipped with a cold-jet modulator and a time-of-flight mass spectrometer (Pegasus 4D, LECO Corp., Saint Joseph, MO, USA). The volatiles were separated via the TG-WAX capillary column (30 m × 0.25 mm, 0.25 μm, Agilent Technologies, Santa Clara, CA, USA) and the DB-17 MS column (2 m × 0.1 mm, 0.1 μm, Agilent Technologies, Santa Clara, CA, USA) as the first- and second-dimension columns, respectively. Helium (99.999%) served as the carrier gas at a flow rate of 1.0 mL/min in splitless mode. The column temperatures adhered to the following protocol: maintaining an initial temperature at 40 °C for one minute, then raised to 160 °C at 3 °C/min. Subsequently, the temperature was raised to 250 °C at a rate of 10 °C/min and held there for 5 min. The offset temperature of the secondary column oven was +5 °C compared to the GC oven, while the modulator temperature had an offset of +15 °C relative to the secondary column oven. After separation via two capillary columns, the analytes were ionized at 70 eV, and the spectra were collected in a mass range of 33–450 amu. The temperature of the ion source was set to 230 °C, while the interface temperature was set to 250 °C. The cold zone temperature was set to −50 °C, and the modulation time was 6 s.

### 2.5. Recombination and Omission Experiments

This analysis was conducted following the established method [19]. A team of twelve healthy participants (eight females and four males) aged 22–35 were chosen for the sensory evaluation. The panelists underwent olfactory training with different flavor standards for at least 15 days. The recombination model of the non-smoked bacon was prepared by mixing all volatile compounds with an FD factor ≥27 at their actual concentration, ultrapure water, and an artificial odorless matrix. Panelists evaluated recombination models and samples and analyzed aroma profiles using a 0-to-10 scale based on the intensity of various aroma attributes.

An omission system was created to confirm the impact of specific volatile compounds on the overall aroma profile. Omission models included all odorants, except one authentic compound, in a recombination model. Panelists evaluated each omission and recombination model through a triangle test. When at least 8 panelists evaluated the omission model to be significantly different from the recombination model, it was considered that the omitted odorant contributed significantly to the aroma profile (*p* < 0.05). When at least nine individuals made the correct choice, a statistically significant difference was observed (*p* < 0.01).

### 2.6. Statistical Analysis

The LECO ChromaTOF 4.5 software (LECO Corp., Saint Joseph, MO, USA) was used for 2D data and picture analysis. Data were analyzed via one-way analysis of variance (ANOVA), and to evaluate significant differences between samples, Duncan’s multiple range test was performed at *p* < 0.05. The data were processed by SPSS software 26.0 (SPSS Inc., Chicago, IL, USA), and the pictures were created by Origin 2022 software (OriginLab Software, Northampton, MA, USA). All results were obtained from three replicates, and they were presented as means ± standard error.

## 3. Results and Discussion

### 3.1. Volatile Compounds in Non-Smoked Bacon Analyzed via GC–IMS and GC × GC–TOFMS

#### 3.1.1. GC–IMS Topographic Plots in Different Non-Smoked Bacon

**Figure 1:**
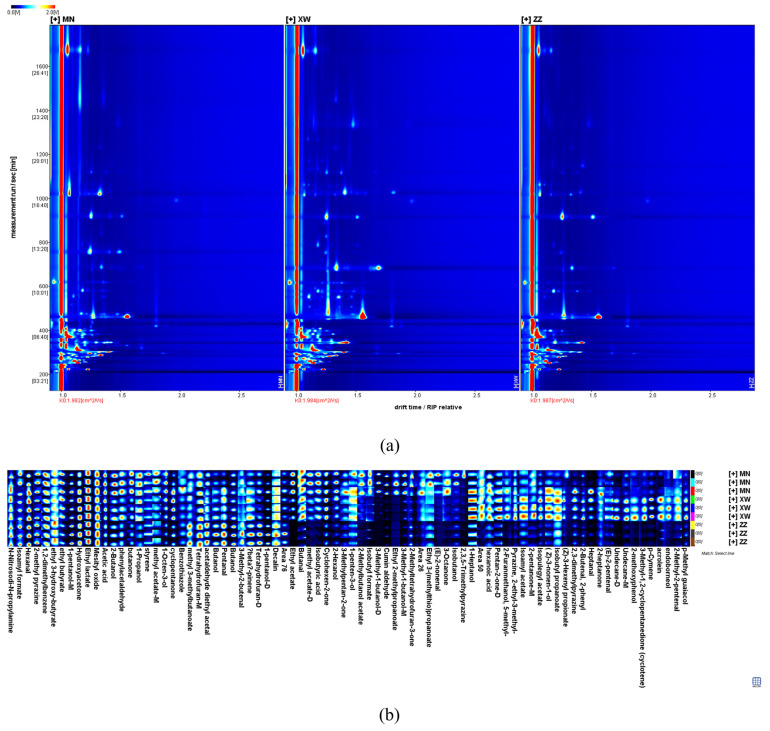
Fingerprints of three non-smoked bacons, obtained with GC–IMS. (**a**) Topographic plots; (**b**) dynamic fingerprints of three non-smoked bacon. While each column represents the same volatile compounds in several samples, each row reflects the signal peak of a single sample. Colors serve as a visual representation of a volatile compound’s content; a brighter color represents greater content.

Figure 1 presents the 2D chromatogram and the fingerprints of volatile compounds in three non-smoked bacons. The drift time and retention time were represented by the X-axis and Y-axis in the 2D top view. Most of the signals appeared in the retention time between 200 s and 500 s and the drift time range of 1.0 to 1.5 riprel (reaction ion peak relative drift times). Figure 1a shows the reaction ion peak (RIP) as a red vertical line on the left, and the standardized drift time is 5.5 ms. Each colored point represents a compound, and the various hues are used to signify concentrations—red denoting more intensity, white denoting absence. The software extracted all volatile compounds from the spectra to develop a fingerprint for intuitively assessing the variations between aromas in the three samples. As shown in Figure 1b, 80 volatile compounds were separated and identified via GC–IMS. Detailed information on these volatiles is presented in Table 1. Each aroma compound was characterized by the retention index and drift time. Due to a limited library database, three substances could not be identified via GC–IMS. Therefore, there is still a need for further improvement in the compounds’ characterization techniques of this method. The remaining 77 volatile compounds included 12 aldehydes, 16 alcohols, 12 ketones, 16 esters, 3 acids, 5 aromatic compounds, 4 alkanes, and 9 other substances. Some volatile compounds form multiple signals including monomers (M) and dimers (D). The formation of different signals from the same compound might be related to the concentration and half-life of the compounds in the drift tube [10]. These compounds had the same retention index, while the drift times were different. Seven volatiles were detected as monomers and dimers, containing 1-pentanol, 3-methyl-1-butanol, butanol, pentan-2-one, methyl acetate, undecane, and tetrahydrofuran. The peak intensities for about half of the aromas were not significantly different between the three samples. Alcohols and esters displayed a higher content in Yunnan bacon, such as isoamyl acetate, ethyl acetate, endoborneol, and (Z)-3-nonen-1-ol. Aldehydes and acids were more abundant in Sichuan bacon, including (E)-2-nonenal, butanal, and acetic acid. Overall, the type and quantity of aroma compounds in Yunnan bacon were higher than those in the other two samples.

#### 3.1.2. Volatile Compounds in Non-Smoked Bacon Identified via GC × GC–TOFMS

**Figure 2:**
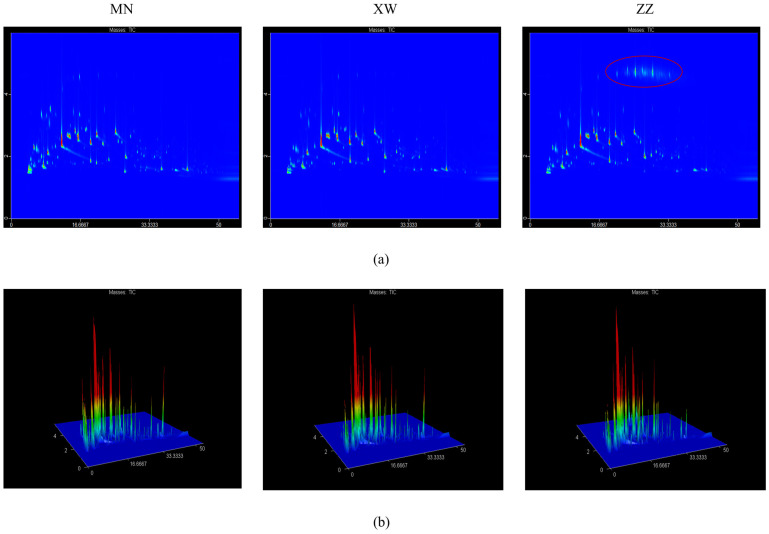
A 2D (**a**) and 3D (**b**) chromatogram of volatile compounds in non-smoked bacon via GC × GC–TOFMS.

The aroma compounds in non-smoked bacon analyzed by GC × GC–TOFMS were visualized in the form of two- and three-dimensional chromatograms. The results of the 2D and 3D chromatograms are shown in Figure 2a,b, respectively. The compounds’ retention time is displayed on the I-axis, while the chemical polarity is represented on the II-axis. It could be found that the peak profiles of the three non-smoked bacon samples were similar, indicating little variation in the species of volatile in different bacon. The hydrocarbon content in the Chongqing bacon was significantly higher than the other two bacon samples, as indicated by the peak in the red circle in Figure 2a. This could be related to the animals’ diet and the conditions in which they are raised. However, the higher content of hydrocarbons did not affect the overall aroma, as their thresholds were too high. The detailed information on GC × GC–TOFMS is shown in Table S1. Three samples of bacon were evaluated, resulting in 188 volatile compounds which were divided into nine groups: 23 hydrocarbons, 35 aldehydes, 31 alcohols, 32 ketones, 21 esters, 13 aromatic compounds, 10 acids, 8 furans, and 15 other compounds. For bacon samples from different regions, a total of 151, 121, and 130 volatiles were detected in Yunnan, Chongqing, and Sichuan bacon, respectively, and their contents were 48,433.69, 35,643.17, and 41,027.38 µg/kg. Chongqing bacon showed the highest hydrocarbon content (4272.73 µg/kg) in the three bacon samples, which was consistent with the results displayed in the 2D and 3D chromatograms. Aldehydes play a key role in overall aroma, and 30, 24, and 22 aldehydes were detected in Yunnan, Chongqing, and Sichuan bacon with contents of 8964.78, 10178.60, and 3858.59 µg/kg, respectively. Although Yunnan bacon had the most types of aldehydes, Chongqing bacon had the highest aldehyde content. This could be attributed to the higher contents of pentanal and hexanal. The highest alcohol content was found in Yunnan bacon, reaching 7841.54 µg/kg. 1-octen-3-ol and ethanol had a higher content in non-smoked bacon. Aldehydes and alcohols comprised the primary volatile compounds found in Yunnan bacon, accounting for 18.51% and 16.19%. For Chongqing bacon, the proportion of aldehydes could reach 28.56%, which were the main volatiles. The aroma of Sichuan bacon was dominated by ketones and alcohols, accounting for 13.52% and 13.35%, respectively. Other compounds, including pyridine, pyrazine, pyrrole, and their derivatives, also play a critical role in meat aroma formation, with the highest content found in Sichuan bacon. Aldehydes and alcohols may be related to the degree of lipid oxidation. It indicates that Chongqing bacon may have the highest degree of oxidation and the most intense aroma. The degree of lipid oxidation in pork is related to its fatty acid composition, induced oxidation, and antioxidant factors.

### 3.2. Key Aroma Compounds in Non-Smoked Bacon Identified via GC–O–MS

AEDA proves to be an efficient method of assessing the impact of individual odor-active compounds on the overall aroma. The aroma in the sample was continuously diluted until assessors could not detect anything. The aroma compounds could be detected with a high FD factor, and they could be identified as key aroma compounds. Due to the complexity of the matrix, the use of chromatographic columns with different polarities can obtain a better separation effect and accurate qualitative analysis. The results of compound identification via GC–O–MS are shown in Table 2. A total of 76 volatile compounds were detected in three non-smoked bacons via GC–O–MS, comprising 49 aroma compounds detected through assessors and mass spectrometry, with 25 volatiles solely identified through mass spectrometry. The other two unknown compounds could only be sniffed by assessors via an olfactometer. These two compounds also have a significant contribution to overall aroma and present mushroom and rice aromas. As shown in Table 2, a total of 13 aldehydes, 10 alcohols, 12 ketones, 13 esters, 6 aromatic compounds, 8 hydrocarbons, 10 acids, and 2 other compounds were detected in the three bacon samples. The concentration and OAV values of each volatile compound are shown in Table 3.

Aldehydes: Aldehydes, both saturated and unsaturated, are a significant contributor to the aroma of meat and meat products due to their low threshold [20]. In this study, Sichuan bacon contained 12 aldehydes; Chongqing and Yunnan bacon had 10 and 9 aldehydes, respectively. Meanwhile, the total concentrations in Yunnan, Sichuan, and Chongqing bacon were 1263.73 ± 14.86, 2818.54 ± 53.99, and 1774.5 ± 42.45 µg/kg. Hexanal, (E)-2-nonenal, and (E,E)-2,4-decadienal were found to be significant in the three bacon samples due to their high FD factor (FD ≥ 27). These compounds are believed to be the primary aldehydes responsible for the aroma of non-smoked bacon. The hexanal concentration was the highest among all volatile compounds in three bacon samples (868.55 ± 41.46, 628.88 ± 28.47, and 1123.21 ± 66.27 µg/kg in Yunnan, Sichuan, and Chongqing bacon). Other aldehydes such as 2-undecenal, octanal, and heptanal were also considered as key aroma compounds in Chongqing and Sichuan bacon. More aldehydes appeared in Sichuan bacon, so nonanal and decanal contributed significantly to the overall aroma of Sichuan bacon. Aldehydes were known to provide a fatty, green, and fresh aroma, and they were derived primarily from the lipid oxidation and Strecker degradation of α-amino acid [21]. Hexanal exhibited the highest FD factor among the aldehyde compounds, and these straight-chain aldehydes primarily originated from the oxidation of both unsaturated and saturated fatty acids [10]. In addition to hexanal, octanal, nonanal, decanal, (E)-2-octenal, (E)-2-nonenal, (E,E)-2,4-decadienal, (E)-2-decenal, 2-undecenal, and heptanal were also straight-chain aldehydes, and all contributed significantly to the overall aroma. These findings align with prior research indicating that hexanal is the predominant aldehyde in dry-cured meat products, including lacón, ham, and chorizo [22]. Linoleic acid may serve as the precursor for the formation of hexanal, heptanal, octanal, and nonanal via the autoxidation of oleic fatty acid [22]. Fatty acids were oxidized into different hydroperoxides via enzymatic oxidation or autooxidation, and these hydroperoxides could degrade into various aldehydes [23]. The aldehydes identified through GC–O–MS were solely straight-chain aldehydes, while a few branched-chain aldehydes were detected through GC–IMS. These aldehydes present fatty and green notes but have an unpleasant rancid aroma at an excessive concentration. (E)-2-nonenal and (E,E)-2,4-decadienal contribute distinct nuances to the aroma profile, with (E)-2-nonenal offering a fatty, waxy scent and (E,E)-2,4-decadienal providing a pungent, fatty aroma. The principal origin of branched-chain aldehydes was the Strecker degradation pathway. Given the high fat content of bacon and the low threshold, aldehydes were the major characteristic volatile compounds of non-smoked bacon. These aldehydes, derived from the oxidation of fatty acids, not only enhance the complexity of the aroma but also evoke a sense of freshness and richness in the bacon’s scent.

Alcohols: A total of ten alcohols were detected in bacon samples, of which eight, five, and eight alcohols were sniffed by assessors in Yunnan, Chongqing, and Sichuan bacon, respectively. Seven alcohols could be detected only via a polar column (SH-Stabilwax-MS). The concentration in Yunnan bacon (652.59 ± 26.12 µg/kg) was also higher than that in Sichuan (577.76 ± 2.6 µg/kg) and Chongqing (344.26 ± 34.04 µg/kg) bacon. Four alcohols were considered as the main contributors to the aroma, including 1-octen-3-ol, 2,3-butanediol, 1-octanol, and 1-hexanol. The content of 1-octen-3-ol was relatively high in bacon alcohols (129.7 ± 5.96, 72.55 ± 1.98, and 101.94 ± 3.28 µg/kg in Yunnan, Chongqing, and Sichuan bacon). Meanwhile, 1-octen-3-ol had the highest OAV among alcohols in bacon, reaching 129.7 ± 5.96, 72.55 ± 1.98, and 101.94 ± 3.28 in Yunnan, Chongqing, and Sichuan bacon, respectively. Other alcohols did not have a significant contribution due to the low FD factor, even 2-ethyl-1-hexanol could not be sniffed. Although alcohols were not the most abundant volatiles, they were also vital to the formation of the bacon aroma [22]. It differs from this study, possibly due to the different raw materials. Previous research indicated that 2,3-butanediol was the main alcohol in the loin and chorizo. 1-Octen-3-ol was the most prominent alcohol found in the three bacon samples and had the highest FD factor in Chongqing and Yunnan bacon. This volatile compound might be the oxidative product of linoleic or other polyunsaturated fatty acids, and it imparts a strong mushroom and earthy aroma to bacon. 1-hexanol had a high FD factor in Yunnan bacon and was considered a hexanal reduction product [23]. In Sichuan bacon, 1-octanol exhibited a high FD factor that was due to the oxidation of oleic acid [24]. These compounds provide herbal and fatty notes and are related to sweet and fruity aromas with certain concentrations [25]. The GC–O–MS analysis detected mostly linear alcohols derived from the oxidation of fatty acids. Conversely, branched alcohols were likely produced through the Strecker degradation of amino acids or the reduction of branched-chain aldehydes [22].

Ketones and esters: Although ketones also play a significant role in the development of the bacon aroma, these compounds were less important than aldehydes and alcohols due to their higher threshold [26]. A total of 12 ketones were determined by GC–O–MS, and 9 ketones could be sniffed by assessors. Among the three bacon samples, six, four, and six ketones could be sniffed in the Yunnan, Chongqing, and Sichuan bacon, respectively. A total of eight, nine, and four ketones were detected by mass spectrometry, and the ketones’ concentrations were 276.82 ± 4.36, 197.15 ± 1.33, and 68.35 ± 3.3 µg/kg in Yunnan, Chongqing, and Sichuan bacon, respectively. Four ketones were identified as having the potential to contribute to the overall aroma due to their high FD factors, including 2,3-octanedione, 5-ethyldihydro-2(3H)-furanone, dihydro-5-pentyl-2(3H)-furanone, and 5-butyldihydro-2(3H)-furanone. However, the results showed that the OAVs of these compounds did not exceed 1, and they could still be sniffed by assessors. Previous research has shown that acetoin is the primary ketone present in the loin, salchichón, and shoulder [22]. This might be related to the material used. Various pathways could generate ketones such as the oxidation of free fatty acids and Maillard reactions [27]. It was worth noting that three of the four key ketones were furanone. 5-ethyldihydro-2(3H)-furanone, dihydro-5-pentyl-2(3H)-furanone, and 5-butyldihydro-2(3H)-furanone—known as γ-caprolactone, γ-nonanolactone, and γ-octanoic lactone—contributed to the overall aroma with a sweet, fatty, and butter aroma. Lactones were mainly from fatty acid oxidation [24].

In general, esters play a vital role in the overall aroma of dry-cured meat due to their low threshold. However, esters were not considered as key volatile compounds in this study because only four esters were sniffed with low FD factors. Although 13 esters were detected via GC–O–MS, only one or two esters could be sniffed in each sample, so esters could have a low influence on bacon samples. The total concentration of esters in Yunnan, Chongqing, and Sichuan bacon was 358.4 ± 29.91, 156.11 ± 1.74, and 15.8 ± 1.53 µg/kg, and the OAV of esters did not exceed 1 except hexanoic acid ethyl ester. The esters seem to be separated well on the non-polar column, because six ketones could only be detected via a non-polar column (SH-Rxi-5Sil MS). The primary origin of esters is the esterification of acids and alcohols in dry-cured meat products [28].

Acids: A total of ten acids were detected in bacon samples, of which nine, eight, and six acids could be sniffed by assessors in Yunnan, Chongqing, and Sichuan bacon, respectively. The acids were abundant in bacon samples, reaching 518.38 ± 16.47, 267.06 ± 7.57, and 118.29 ± 0.28 µg/kg in Yunnan, Chongqing, and Sichuan bacon, respectively. However, the OAV of the acids did not exceed 1 except 3-methyl-butanoic acid. For AEDA tests, 3-methyl-butanoic acid and hexanoic acid had higher FD factors and could be considered potential key aroma compounds in non-smoked bacon. This finding contrasts with prior research which pointed to acetic acid as the primary source of organic acid in meat products, notably sausages [23]. Acetic acid is usually generated in meat products that have a fermentation stage, so it could come from the microorganism [29]. Straight-chain acids typically originate from the hydrolysis of fatty acids, while branched acids can be produced by the oxidation of their respective Strecker aldehydes [30]. Therefore, 3-methyl-butanoic acid could originate from the oxidation of 3-methyl-butanal. Another study suggests that aminotransferase could be involved in the degradation of isoleucine and leucine, resulting in the production of 3-methyl-butanoic acid [31]. 3-methyl-butanoic acid contributes to the feet and cheese aroma, while hexanoic acid and heptanoic acid provide a sweaty and sour aroma. Previous investigations revealed that organic acids could exhibit pleasant aromas in minimal amounts [31]. Other organic acids might partially contribute to the overall aroma of bacon, including acetic acid, butanoic acid, pentanoic acid, octanoic acid, nonanoic acid, and decanoic acid.

Others: A total of eight hydrocarbons, six aromatic compounds, and two other compounds were detected in bacon via GC–O–MS. Hydrocarbons have minimal impact on the overall aroma of meat products because of their high threshold [32]. Hydrocarbons including 2,6,10-trimethyl-dodecane, 3-methyl-tridecane, tetradecane, pentadecane, and hexadecane were detected via MS, but none could be sniffed by assessors in the three samples. Alkanes with carbon chains of less than ten carbons may originate from lipid oxidation, whereas other hydrocarbons may accumulate in the fat from feeding [22]. Aromatic compounds could be sniffed by assessors in the three samples but not the key aroma compounds. Benzaldehyde, butylated hydroxytoluene, and p-cresol might contribute to the overall aroma of Yunnan, Chongqing, and Sichuan bacon, respectively. 2-Pentyl-furan was commonly found in meat products and provided a fruity aroma. This compound was derived from lipid oxidation or a Maillard reaction but has little effect on the aroma of bacon in this study [21].

The results indicate that the differences in aroma compounds between different types of bacon are minimal. The variations in flavor primarily stem from differences in the content of each substance. Aldehydes and alcohols are produced from lipid oxidation, while ketones and esters may result from the Maillard reaction. Acids may be derived from microbial metabolism. The variation in volatile content may be attributed to differences in processing and raw material selection, the fatty acid composition of pork from various regions, pig feeding methods and growing environments, and the presence of induced oxidative and antioxidative factors in pork. These factors may be the primary reason for the differences in aroma.

### 3.3. Comparison of Volatile Compounds in Non-Smoked Bacon via GC–O–MS, GC × GC–TOFMS, and GC–IMS

**Figure 3:**
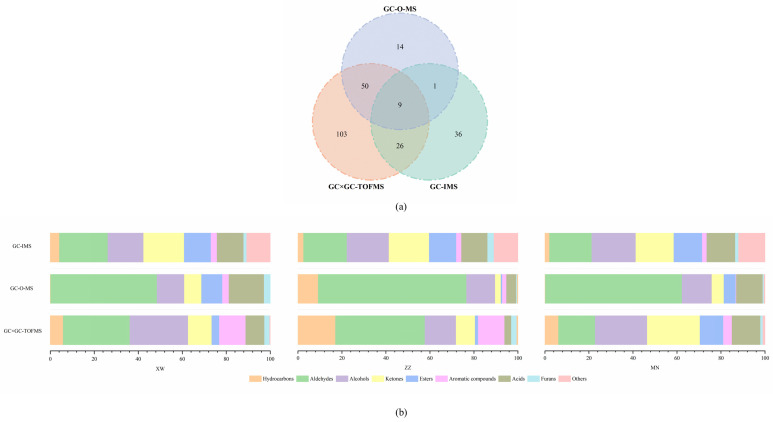
Comparison of Venn diagrams for extraction of volatile compounds by GC–O–MS, GC × GC–TOFMS, and GC–IMS (**a**); comparison of quantities of various volatile compounds extracted from non-smoked bacon by GC–O–MS, GC × GC–TOFMS, and GC–IMS (**b**).

The distinctions among the aroma compounds identified by GC–O–MS, GC × GC–TOFMS, and GC–IMS are illustrated in Figure 3. A total of 239 volatiles were identified in the three non-smoked bacon samples, and 74, 188, and 72 volatiles were detected via GC–O–MS, GC × GC–TOFMS, and GC–IMS, respectively. However, only nine aroma compounds could be detected by all three methods, including hexanal, heptanal, 1-pentanol, acetic acid, 1-octen-3-ol, 1-heptanol, methyl isovalerate, dihydro-5-propyl-2(3H)-furanone, and hexanoic acid. This might be attributed to the different extraction, separation, and detection methods used in the three methods. SPME was applied to extract volatile compounds in GC–O–MS and GC × GC–TOFMS, while GC–IMS just injected 0.5 mL headspace gas into the instrument via an 85 °C heated syringe. GC × GC–TOFMS separated volatiles via two columns with different polarities so that analytes with similar physical and chemical properties could be separated better than GC–O–MS. GC–IMS ionized the volatiles in an ion transfer tube and migrated to a Faraday disk for secondary separation after initial separation via a column. The mass spectrometer identifies volatiles according to the mass-to-charge ratio (*m*/*z*) of the ionic fragments of compounds, even though TOFMS has a fast scanning rate and is capable of handling tiny peaks flowing quickly [33]. However, GC–IMS analyzed the compounds based on differences in the mobility of gas-phase ions [11]. Therefore, the types and contents of compounds detected by different methods will be different. Only by combining various methods to characterize the aroma of bacon can more comprehensive results be obtained. In this study, even with the same extraction method, 79.73% of volatiles detected by GC–O–MS could also be identified via GC × GC–TOFMS. However, half of the volatiles detected by GC–IMS were not identified via the other two methods. As shown in Figure 3b, the sensitivity of different substances varies depending on the detection method used. GC–O–MS has more advantages in aldehyde detection, while GC × GC–TOFMS has a higher sensitivity to hydrocarbons and alcohols. This may be due to the fact that the materials used in the volatile extraction process have a higher affinity for aldehydes and alcohols, and the chromatographic columns used are polar columns, which have a higher degree of separation for such substances. This leads to the higher sensitivity of these two methods for aldehydes and alcohols. As a result, bacon aroma profiles detected by both methods may tend to be more fatty or even rancid. If there is less extraction or a poorer separation of esters, more sweetness will be lost in the aroma profile. The sensitivity of GC–IMS to various compounds was relatively uniform, which could be attributed to the lack of selectivity in the volatile extraction process. Also, the limited database restricts the species of compounds that can be used. This can lead to the possibility that important compounds may be missed, and the aroma profile of the bacon may not be truly reflected. In addition, most of the alcohols, ketones, and esters detected by GC–IMS were not characterized via the other two methods. The results of this research were consistent with a previous study, which illustrated that only 3.50% of total aroma compounds in grilled lamb shashliks could be detected by all three methods (GC–MS, GC × GC–TOFMS, and GC–IMS) [34]. In general, GC–IMS is better at the detection of trace aroma compounds and their comparison between different samples. Meanwhile, GC × GC–TOFMS provided a better separation effect and extreme sensitivity. However, the human sense of smell is a more sensitive detector, and the methods combined with it led to more realistic results. GC–O–MS could combine instrumental detection with human senses and had better quantitative capability than the other two methods [15]. A combination of all three methods can be used to maximize the understanding of all the aroma compounds in non-smoked bacon. At the same time, it can provide a reference extraction and detection method for studying the target compounds in future research.

### 3.4. Aroma Recombination and Omission Studies of the Non-Smoked Bacon

**Figure 4:**
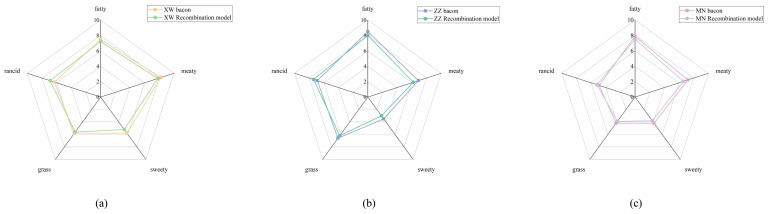
Aroma profiles of non-smoked bacon compared with aroma recombination model. (**a**) Recombination model of XW bacon; (**b**) Recombination model of ZZ bacon; (**c**) Recombination model of MN bacon.

Recombination experiments are commonly used to validate whether key aroma compounds were accurately identified via AEDA and OAV methods [31]. In molecular sensory science, recombination experiments were necessary to accurately simulate the aroma. In this study, the sensory panel discussed the descriptors for the aroma profile of non-smoked bacon as “fatty”, “meaty”, “sweety”, “rancid”, and “grass”. The results of GC–O–MS experiments showed that 12, 9, and 15 volatile compounds had FD factors ≥ 27 in Yunnan, Chongqing, and Sichuan bacon, respectively. Therefore, the recombination model of each bacon sample containing these odorants (except for the unknown volatiles) has concentrations that have been calibrated via the external standard method. The data are shown in Table 4, revealing the high linearity of the aroma compounds as indicated by the correlation coefficients (R^2^ > 0.99). Figure 4 shows that the aroma profile of the recombination models was similar to their original bacon sample. Among these five aroma notes, meaty and fatty were dominate. The rancid aroma also played a vital role in Yunnan and Chongqing bacon. Meanwhile, Yunnan bacon obtained the highest sweety score, which could be attributed to the higher ester content than the other two bacons. The total sensory evaluation scores for five aroma attributes exceeded 75% of the original sample, indicating a good simulation of the aroma characteristics of the bacon sample. However, the rancid aroma in the recombination model was higher than in the original sample in all three bacons. The excessive addition of aldehydes might be the reason for this result.

Omission tests were performed to determine the impact of individual odorants on the overall aroma and to identify active odorants. As shown in Table 5, 12, 9, and 15 volatile compounds in Yunnan, Chongqing, and Sichuan bacon were selected for omission experiences via the triangulation test. A total of eight, eight, and nine aroma compounds were significantly different from the recombination model after omission in Yunnan, Chongqing, and Sichuan bacon, respectively. These compounds were identified as the key aroma compounds in their respective samples and made a notable impact on the overall aroma. Among these compounds, hexanal, (E,E)-2,4-decadienal, 1-octen-3-ol, dihydro-5-pentyl-2(3H)-furanone, and 3-methyl-butanoic acid were identified as the key aroma compounds shared by all three bacon samples. Meanwhile, 2,3-octanedione, hexanoic acid, and 2-undecenal were considered the key aroma compounds in two bacon samples. For Yunnan and Chongqing bacon, 1-hexanol and (E)-2-nonenal were identified as the key aroma compounds unique to their respective sample. Similarly, octanal and 1-octanol were found to be the key compounds only in the Sichuan bacon sample. Other compounds, including 2,3-butanediol, pentanoic acid, heptanoic acid, nonanal, decanal, heptanal, and 5-ethyldihydro-2(3H)-furanone, had no significant differences in the recombination model after omission. This result suggested that these volatile compounds had little impact on the overall aroma, or their contribution was overshadowed by other compounds. The sensory evaluation results not only validate the identified key aroma compounds but also support the effectiveness of recombination models in accurately simulating the aroma characteristics of bacon samples. Therefore, the key aroma compounds of non-smoked bacon were hexanal, (E,E)-2,4-decadienal, 1-octen-3-ol, dihydro-5-pentyl-2(3H)-furanone, and 3-methyl-butanoic acid. Recombination and omission experiments have been used to identify key aroma compounds and their contents in bacon from different regions. This provides effective molecular information for the quality evaluation and geographic differentiation of non-smoked bacon. The variations in the aroma of non-smoked bacon primarily resulted from the quantity of different volatiles, while the differences in the categories of volatile compounds were trivial.

## 4. Conclusions

In this study, GC–O–MS, GC × GC–TOFMS, and GC–IMS were simultaneously employed to analyze and identify odor-active compounds in non-smoked bacon. Among three bacon samples, 239 volatiles were identified, and 74, 188, and 72 volatiles were detected via GC–O–MS, GC × GC–TOFMS, and GC–IMS, respectively. GC × GC–TOFMS and GC–IMS could provide high resolution and trace detection capabilities but could not be combined with human senses. GC–O–MS improved this defect and had more advantages in the quantification of volatiles. After recombination and omission tests, hexanal, (E,E)-2,4-decadienal, 1-octen-3-ol, dihydro-5-pentyl-2(3H)-furanone, and 3-methyl-butanoic acid were considered the key aroma compounds in non-smoked bacon. Seven volatiles were considered contributors in the formation of aromas from different regions, including 2,3-octanedione, hexanoic acid, 2-undecenal, 1-hexanol, (E)-2-nonenal, octanal, and 1-octanol. Instead of the type, the content of volatiles was the primary factor affecting the aroma of non-smoked bacon from Yunnan, Sichuan, and Chongqing bacon. These findings can provide valuable molecular information for enhancing aroma quality, assessing product quality, and distinguishing non-smoked bacon by geographic origin. Future studies could investigate the impact of important steps in bacon aroma formation on key aroma compounds.

## Figures and Tables

**Table 1:** Volatile compounds identified by GC-IMS in non-smoked bacon.

No.	Compounds	CAS	Formula	MW	RI	Rt	Dt	Comment	Intensity		
									MN	XW	ZZ
	Aldehydes										
1	2-Phenyl-2-butenal	4411-89-6	C_10_H_10_O	146.2	1274	1120.272	1.25211	null	1032.07 ± 212.84 ^a^	2227.82 ± 247.86 ^b^	918.65 ± 165.77 ^a^
2	Cumin aldehyde	122-03-2	C_10_H_12_O	148.2	1248.5	1033.13	1.32881	null	7490.28 ± 528.96 ^b^	1996.99 ± 117.78 ^a^	1874.56 ± 280.68 ^a^
3	(E)-2-nonenal	18829-56-6	C_9_H_16_O	140.2	1178.1	758.443	1.40708	null	1203.76 ± 300.53 ^b^	512.86 ± 46.16 ^a^	403.28 ± 25.47 ^a^
4	(E)-2-pentenal	1576-87-0	C_5_H_8_O	84.1	1135.5	619.551	1.35549	null	567.27 ± 163.38 ^b^	879.12 ± 51.65^c^	314.82 ± 38.23 ^a^
5	Hexanal	66-25-1	C_6_H_12_O	100.2	1073.2	463.893	1.56003	null	14,982.01 ± 7895.8 ^a^	19,343.69 ± 2755.72 ^a^	14,002.65 ± 1228.08 ^a^
6	Phenylacetaldehyde	122-78-1	C_8_H_8_O	120.2	1026.6	399.105	1.25636	null	1268.42 ± 176.14 ^b^	1030.65 ± 252.25 ^ab^	678.21 ± 100.88 ^a^
7	Pentanal	110-62-3	C_5_H_10_O	86.1	986.5	346.932	1.42372	null	2556.28 ± 2158.24 ^a^	4105.63 ± 717.16 ^a^	4489.65 ± 612.7 ^a^
8	Acrolein	107-02-8	C_3_H_4_O	56.1	841.5	272.365	1.05565	null	340.91 ± 101.35 ^a^	1521.76 ± 220.57 ^b^	338.19 ± 76.28 ^a^
9	3-Methyl-2-butenal	107-86-8	C_5_H_8_O	84.1	770.2	243.259	1.08451	null	372.47 ± 60.14 ^ab^	295.76 ± 30.88 ^a^	426.43 ± 50.75 ^b^
10	Butanal	123-72-8	C_4_H_8_O	72.1	882.5	289.099	1.10631	null	792.55 ± 79.7 ^b^	689.4 ± 42.25 ^b^	161.43 ± 1.86 ^a^
11	Heptanal	111-71-7	C_7_H_14_O	114.2	1159.5	685.1	1.69677	null	2540.25 ± 3625.8 ^a^	3259.79 ± 1265.18 ^a^	641.64 ± 299.15 ^a^
12	2-Methyl-2-pentenal	623-36-9	C_6_H_10_O	98.1	1124.3	591.599	1.16224	null	217.01 ± 77.48 ^b^	342.8 ± 12.04^c^	101.6 ± 23.42 ^a^
	Alcohols										
13	1-Octen-3-ol	3391-86-4	C_8_H_16_O	128.2	1436.4	1675.329	1.15976	null	5806.28 ± 402.67 ^a^	4127.67 ± 875.31 ^b^	2303.56 ± 278.74^c^
14	1-Pentanol	71-41-0	C_5_H_12_O	88.1	1219.3	921.36	1.25681	M	6676.16 ± 2342.53 ^a^	6574.05 ± 287.53 ^a^	7866.56 ± 717.7 ^a^
15	1-Pentanol	71-41-0	C_5_H_12_O	88.1	1218.3	917.572	1.50882	D	2368.67 ± 1394.37 ^a^	2171.94 ± 167.02 ^a^	3668.55 ± 777.23 ^a^
16	3-Methyl-1-butanol	123-51-3	C_5_H_12_O	88.1	1177.1	754.654	1.4916	D	2943.07 ± 318.44 ^b^	921.92 ± 60.54 ^a^	722.77 ± 80.81 ^a^
17	3-Methyl-1-butanol	123-51-3	C_5_H_12_O	88.1	1178.1	758.443	1.24116	M	6600.43 ± 595.61^c^	2273.48 ± 387.36 ^b^	1421.81 ± 199.14 ^a^
18	Butanol	71-36-3	C_4_H_10_O	74.1	1120.1	581.135	1.18272	M	1700.83 ± 135.33 ^a^	1904.22 ± 193.6 ^ab^	2189.02 ± 161.72 ^b^
19	Butanol	71-36-3	C_4_H_10_O	74.1	1120.9	582.965	1.37911	D	338.45 ± 107.93 ^a^	458.52 ± 71.9 ^ab^	560.22 ± 120.27 ^b^
20	1-Penten-3-ol	616-25-1	C_5_H_10_O	86.1	1120.3	581.469	1.35116	null	401.68 ± 138.9 ^a^	439.1 ± 29.41 ^a^	290.78 ± 26.13 ^a^
21	Isobutanol	78-83-1	C_4_H_10_O	74.1	1078.7	477.547	1.16906	null	856.95 ± 523.29 ^a^	339.51 ± 76.92 ^a^	482.18 ± 19.13 ^a^
22	1-Propanol	71-23-8	C_3_H_8_O	60.1	1029.1	402.379	1.1083	null	1826.39 ± 76.15^c^	1659.05 ± 56.09 ^b^	869.12 ± 61.18 ^a^
23	2-Butanol	78-92-2	C_4_H_10_O	74.1	1028.8	401.97	1.16018	null	2713.11 ± 51.24 ^b^	2139.17 ± 391.12 ^a^	1657.38 ± 172.27 ^a^
24	1-Heptanol	111-70-6	C_7_H_16_O	116.2	985.2	345.35	1.3895	null	1335.95 ± 670.17 ^ab^	1987.96 ± 73.36 ^b^	1038.47 ± 149.55 ^a^
25	2-Hexanol	626-93-7	C_6_H_14_O	102.2	803.9	257.034	1.28093	null	160.09 ± 4.09 ^b^	131.02 ± 27.25 ^b^	64.22 ± 4.68 ^a^
26	5-Methyl-2-furanmethanol	3857-25-8	C_6_H_8_O_2_	112.1	952	325.69	1.26008	null	286.23 ± 84.12 ^b^	299.82 ± 57.26 ^b^	72.9 ± 23.44 ^a^
27	Endoborneol	507-70-0	C_10_H_18_O	154.3	1167.5	716.542	1.21743	null	200.18 ± 55.23 ^a^	388.43 ± 47.52 ^b^	123.06 ± 9.38 ^a^
28	(Z)-3-nonen-1-ol	10340-23-5	C_9_H_18_O	142.2	1159.5	685.078	1.43546	null	517.18 ± 303.14 ^ab^	808.96 ± 28.35 ^b^	242.53 ± 44.49 ^a^
	Ketones										
29	Cyclopentanone	120-92-3	C_5_H_8_O	84.1	1161.2	691.808	1.34058	null	4223.43 ± 2787.21 ^a^	5886.26 ± 893.38 ^a^	3146.04 ± 705.27 ^a^
30	2-Heptanone	110-43-0	C_7_H_14_O	114.2	1158.7	681.747	1.26103	null	1542.72 ± 792.29 ^a^	1826.45 ± 93.7 ^a^	1044.75 ± 157.83 ^a^
31	3-Methylpentan-2-one	565-61-7	C_6_H_12_O	100.2	1077.5	474.513	1.47702	null	999.33 ± 190.33^c^	716.47 ± 21.76 ^b^	467.12 ± 72.18 ^a^
32	3-Methyl-1,2-cyclopentanedione (cyclotene)	80-71-7	C_6_H_8_O_2_	112.1	1015.9	385.194	1.13993	null	291.49 ± 85.31 ^a^	1019.99 ± 99.44 ^b^	227.74 ± 26.71 ^a^
33	2-Pentanone	107-87-9	C_5_H_10_O	86.1	989.7	351.179	1.12415	M	830.23 ± 46.12 ^b^	839.11 ± 326.82 ^b^	293.69 ± 8.87 ^a^
34	2-Methyltetrahydrofuran-3-one	3188-00-9	C_5_H_8_O_2_	100.1	1257.9	1065.334	1.07367	null	5105.7 ± 1042.13 ^b^	2613.35 ± 225.66 ^a^	3372.73 ± 158.06 ^a^
35	Cyclohexen-2-one	930-68-7	C_6_H_8_O	96.1	911.2	302.805	1.40278	null	3416.98 ± 352.54 ^b^	4084.01 ± 368.03^c^	1841.85 ± 95.67 ^a^
36	3-Octanone	106-68-3	C_8_H_16_O	128.2	964.2	332.578	1.31219	null	268.08 ± 67.69 ^b^	98.71 ± 19.71 ^a^	48.42 ± 11.3 ^a^
37	Pentan-2-one	107-87-9	C_5_H_10_O	86.1	984.7	344.697	1.36494	D	1467.15 ± 522.78 ^b^	2181.73 ± 75.49^c^	799.35 ± 124.64 ^a^
38	Butanone	78-93-3	C_4_H_8_O	72.1	899.3	296.139	1.24164	null	2566.49 ± 38.88^c^	1709.41 ± 136.73 ^b^	1488.81 ± 107.48 ^a^
39	Hydroxyacetone	116-09-6	C_3_H_6_O_2_	74.1	725	224.818	1.22641	null	2598.7 ± 467.46 ^a^	3507.62 ± 29.96 ^b^	3153.34 ± 305.13 ^ab^
40	Mesityl oxide	141-79-7	C_6_H_10_O	98.1	813.9	261.078	1.11623	null	6729.15 ± 146.9 ^b^	5840.38 ± 233.57 ^a^	6691.56 ± 372.75 ^b^
	Esters										
41	(Z)-3-Hexenyl propionate	33467-74-2	C_9_H_16_O_2_	156.2	1372.7	1457.474	1.35699	null	1629.29 ± 807.64 ^a^	1816.99 ± 240.06 ^a^	978.89 ± 39.23 ^a^
42	Ethyl 3-(methylthio)propanoate	13327-56-5	C_6_H_12_O_2_S	148.2	1078.1	476.03	1.21459	null	363.44 ± 213.61 ^a^	176.85 ± 48.87 ^a^	251.31 ± 11.8 ^a^
43	Isoamyl formate	110-45-2	C_6_H_12_O_2_	116.2	1070.5	457.066	1.27082	null	1879.94 ± 566.87 ^a^	1564.48 ± 122.78 ^a^	2108.43 ± 177.37 ^a^
44	Isobutyl formate	542-55-2	C_5_H_10_O_2_	102.1	964.7	332.851	1.20094	null	892.69 ± 133.04 ^b^	349.92 ± 82.16 ^a^	205.98 ± 48.05 ^a^
45	Ethyl 2-methylpropanoate	97-62-1	C_6_H_12_O_2_	116.2	960.8	330.616	1.55549	null	603.01 ± 117.39 ^b^	215.44 ± 24.83 ^a^	203.9 ± 35.67 ^a^
46	Methyl 3-methylbutanoate	556-24-1	C_6_H_12_O_2_	116.2	986.7	347.242	1.19675	null	965.62 ± 81.79 ^b^	759.79 ± 126.31 ^a^	1408.24 ± 19.67^c^
47	Ethyl acetate	141-78-6	C_4_H_8_O_2_	88.1	879.1	287.696	1.33464	null	5325.65 ± 20.87 ^b^	2615.38 ± 92.44^c^	217.77 ± 43.87 ^a^
48	2-Methylbutanol acetate	624-41-9	C_7_H_14_O_2_	130.2	892.7	293.251	1.28894	null	642.69 ± 14.2^c^	298.6 ± 22.32 ^b^	149.65 ± 11.16 ^a^
49	Methyl acetate	79-20-9	C_3_H_6_O_2_	74.1	826.3	266.144	1.19194	D	414.05 ± 42.93^c^	255.97 ± 28.37 ^b^	103.66 ± 6.91 ^a^
50	Methyl acetate	79-20-9	C_3_H_6_O_2_	74.1	829	267.255	1.0308	M	380.4 ± 32.49 ^a^	267.37 ± 22.53 ^b^	278.27 ± 43.4 ^b^
51	Isobutyl propanoate	540-42-1	C_7_H_14_O_2_	130.2	869.3	283.697	1.28012	null	683.28 ± 374.99 ^a^	1250.82 ± 32.77 ^b^	845.53 ± 110.56 ^ab^
52	Ethyl 3-hydroxy-butyrate	5405-41-4	C_6_H_12_O_3_	132.2	906.7	300.307	1.17244	null	349.79 ± 50.55 ^a^	317.37 ± 20.64 ^a^	453.18 ± 54.04 ^b^
53	Ethyl butyrate	105-54-4	C_6_H_12_O_2_	116.2	803.3	256.767	1.2075	null	778.07 ± 48.28 ^b^	874.46 ± 92.28 ^b^	502.39 ± 1.21 ^a^
54	Isoamyl acetate	123-92-2	C_7_H_14_O_2_	130.2	1134.5	617.134	1.31495	null	204.49 ± 53.06 ^a^	415.03 ± 16.05 ^b^	168.47 ± 15.34 ^a^
55	Isopulegyl acetate	89-49-6	C_12_H_20_O_2_	196.3	1274.2	1120.946	1.3833	null	572.72 ± 171.61 ^a^	1079.21 ± 42.98 ^b^	380.05 ± 67.17 ^a^
56	Ethyl lactate	97-64-3	C_5_H_10_O_3_	118.1	791.1	251.81	1.1407	null	6738.69 ± 342.72 ^a^	7724.25 ± 62.49 ^b^	6978.09 ± 347.95 ^a^
	Acids										
57	Acetic acid	64-19-7	C_2_H_4_O_2_	60.1	1440.3	1688.59	1.05645	null	19,402.19 ± 1032.21 ^a^	16,626.92 ± 906.72 ^b^	13,619.52 ± 623.04^c^
58	hexanoic acid	142-62-1	C_6_H_12_O_2_	116.2	987.1	347.826	1.29479	null	1455.95 ± 499.65 ^b^	1904.71 ± 138.14 ^b^	730.86 ± 101.78 ^a^
59	Isobutyric acid	79-31-2	C_4_H_8_O_2_	88.1	822.5	264.589	1.15426	null	1677.81 ± 63.41^c^	1361.61 ± 71.74 ^b^	323.87 ± 23.33 ^a^
	Aromatic compounds										
60	Styrene	100-42-5	C_8_H_8_	104.2	1219.3	921.36	1.41021	null	1167.08 ± 58.58 ^b^	937.25 ± 62.2 ^a^	1107.18 ± 93.73 ^b^
61	2-Methoxyphenol	90-05-1	C_7_H_8_O_2_	124.1	1091.9	510.544	1.10837	null	101.31 ± 50.95 ^a^	318.66 ± 94.36 ^b^	51.67 ± 7.43 ^a^
62	p-Cymene	99-87-6	C_10_H_14_	134.2	1014.1	382.918	1.30901	null	467.69 ± 14.37 ^b^	781.44 ± 92.72^c^	192.11 ± 18.51 ^a^
63	1,2-Dimethylbenzene	95-47-6	C_8_H_10_	106.2	899.7	296.361	1.06367	null	933.37 ± 63.1 ^a^	859.72 ± 46.46 ^a^	845.01 ± 53.28 ^a^
64	p-Methyl guaiacol	93-51-6	C_8_H_10_O_2_	138.2	1186.3	791.085	1.18367	null	887.62 ± 85.03 ^a^	1656.03 ± 168.95 ^b^	669.05 ± 71.28 ^a^
	Hydrocarbons										
65	Undecane	1120-21-4	C_11_H_24_	156.3	1110.3	556.44	1.10442	M	491.55 ± 222.74 ^b^	1879.18 ± 371.65^c^	176.03 ± 33.43 ^a^
66	Undecane	1120-21-4	C_11_H_24_	156.3	1108.8	552.781	1.35425	D	681.7 ± 164.06 ^a^	1813.28 ± 136.3 ^b^	376.98 ± 22.6 ^a^
67	β-pinene	127-91-3	C_10_H_16_	136.2	1120.1	581.135	1.22125	null	457.98 ± 31.59 ^a^	621.45 ± 51.86 ^b^	578.82 ± 105.57 ^ab^
68	Decalin	91-17-8	C_10_H_18_	138.3	1071.6	459.848	1.34063	null	1978.78 ± 394.92 ^a^	2479.8 ± 56.82 ^a^	1993.26 ± 221.56 ^a^
	Others										
69	2,3,5-Trimethylpyrazine	14667-55-1	C_7_H_10_N_2_	122.2	1373.8	1461.263	1.15976	null	8770.03 ± 8081.31 ^a^	3253.72 ± 593.02 ^a^	2099.61 ± 261.55 ^a^
70	2,3-Dimethylpyrazine	5910-89-4	C_6_H_8_N_2_	108.1	1339.4	1343.81	1.48064	null	2281.48 ± 1902.79 ^a^	3182.45 ± 628.12 ^a^	1571 ± 233.44 ^a^
71	N-Nitrosodi-N-propylamine	621-64-7	C_6_H_14_N_2_O	130.2	1076	470.72	1.27082	null	6488.57 ± 1717.04 ^a^	7955.04 ± 546.09 ^a^	7247.1 ± 711.97 ^a^
72	Pyrazine, 2-ethyl-3-methyl-	15707-23-0	C_7_H_10_N_2_	122.2	1002.1	367.272	1.17818	null	805.62 ± 174.83 ^b^	1120.47 ± 171.76^c^	272.21 ± 34 ^a^
73	Tetrahydrofuran	109-99-9	C_4_H_8_O	72.1	859.5	279.697	1.22481	D	1710.09 ± 353.57 ^a^	1442.76 ± 253.38 ^a^	2693.38 ± 318.37 ^b^
74	Tetrahydrofuran	109-99-9	C_4_H_8_O	72.1	856.8	278.587	1.06447	M	773.17 ± 51.78 ^a^	726.02 ± 35.79 ^a^	863.56 ± 10.45 ^b^
75	Acetaldehyde diethyl acetal	105-57-7	C_6_H_14_O_2_	118.2	866.6	282.586	1.13101	null	634.67 ± 56.13 ^a^	857.43 ± 36.96 ^b^	933.69 ± 43.44 ^b^
76	2-Methyl pyrazine	109-08-0	C_5_H_6_N_2_	94.1	787.1	250.147	1.07088	null	604.86 ± 29.1 ^b^	508.55 ± 22.23 ^a^	637.94 ± 13.92 ^b^
77	Benzothiazole	95-16-9	C_7_H_5_NS	135.2	1243.9	1017.518	1.16136	null	1347.67 ± 407.86 ^b^	756.5 ± 34.05 ^a^	715.51 ± 69.66 ^a^

MW: molecular mass; RI: retention index; Rt: retention time; Dt: drift time; M: monomers; D: dimers. Different letters in the same row indicate significant differences (*p* < 0.05).

**Table 2:** The volatile compounds in non-smoked bacon identified via GC–O–MS.

No.	Threshold	Compounds	SH-Rxi-5Sil MS		SH-Stabilwax-MS		Identification Methods	Odor	CAS	FD Factors		
			Literature RI	Calculated RI	Literature RI	Calculated RI				XW	ZZ	MN
		Aldehydes										
1	4	Hexanal	800	798	1083	1080	MS, RI, O, S	Green, grass	66-25-1	81	243	27
2	0.7	Octanal	1003	987	1289	1279	MS, RI, O, S	Fresh, fatty	124-13-0	3	9	27
3	−	(Z)-2-heptenal	958	939	1322	1313	MS, RI, O	Green	57266-86-1	−	1	1
4	8	Nonanal	1104	1095	1391	1384	MS, RI, O, S	Fatty, fresh	124-19-6	1	1	81
5	4	(E)-2-octenal	1060	1046	1429	1419	MS, RI, O, S	Leaf, herbal	2548-87-0	9	9	9
6	3	Decanal	1206	1191	1498	1494	MS, RI, O, S	Floral, orange	112-31-2	3	−	27
7	0.25	(E)-2-nonenal	1162	1152	1534	1533	MS, RI, O, S	Cucumber, green	18829-56-6	27	81	9
8	0.1	(E,E)-2,4-nonadienal	1188	1198	1700	−	MS, RI, S	−	5910-87-2	−	−	−
9	0.2	(E,E)-2,4-decadienal	1317	1309	1811	1824	MS, RI, O, S	Cucumber, oily	25152-84-5	81	27	81
10	3	Heptanal	901	883	1184	1175	MS, RI, O, S	Green, herbal	111-71-7	−	9	27
11	0.3	(E)-2-decenal	1263	1249	1644	1648	MS, RI, O, S	Mushroom, green	3913-81-3	−	9	9
12	1.4	2-Undecenal	1367	1352	1751	1760	MS, RI, O, S	Peel, fresh	2463-77-6	−	27	27
13	0.04	(E,Z)-2,4-decadienal	1295	−	1754	1773	MS, RI, O	Fatty, green	25152-83-4	−	−	3
		Alcohols										
14	150.2	1-Pentanol	765	774	1250	1247	MS, RI, O	Fusel, balsam	71-41-0	1	1	−
15	1	1-Octen-3-ol	980	964	1450	1448	MS, RI, O, S	Earthy, green	3391-86-4	243	243	81
16	25,482	2-Ethyl-1-hexanol	1030	−	1491	1486	MS, RI	−	104-76-7	−	−	−
17	−	2,3-Butanediol	788	−	1543	1538	MS, RI, O, S	Creamy, buttery	513-85-9	27	1	81
18	110	1-Octanol	1071	−	1557	1558	MS, RI, O, S	Green, orange	111-87-5	3	9	27
19	20	(E)-2-octen-1-ol	1067	−	1614	1616	MS, RI, O	Green, fatty	18409-17-1	−	1	1
20	−	Isopinocarveol	118	−	1646	1645	MS, RI, O	Woody, balsam	6712-79-4	1	−	−
21	5.6	1-Hexanol	868	−	1355	1344	MS, RI, O, S	Sweet, green	111-27-3	27	−	−
22	3	1-Heptanol	970	954	1453	1447	MS, RI, O	Leafy, musty	111-70-6	−	−	1
23	140	Phenylethyl alcohol	1116	−	1906	1924	MS, RI, O	Floral, dried	60-12-8	−	−	1
		Ketones										
24	40	5-Nonanone	1073	−	1334	1329	MS, RI	−	502-56-7	−	−	−
25	110	2,3-Octanedione	984	−	1335	1326	MS, RI, O, S	Asparagus, cortex	585-25-1	27	27	1
26	100	(E,E)-3,5-octadien-2-one	1073	1068	1570	1571	MS, RI, O	Fruity, green	30086-02-3	1	−	9
27	65	Acetophenone	1065	−	1647	1657	MS, RI, O	Almond, sweet	98-86-2	3	−	−
28	14	Acetoin	713	−	1284	1278	MS, RI, O	Milky, buttery	513-86-0	−	3	−
29	50.2	2-Octanone	990	−	1287	1274	MS, RI, O	Earthy, weedy	111-13-7	−	−	1
30	−	(E)-3-octen-2-one	1033	1025	1396	1390	MS, RI	−	18402-82-9	−	−	−
31	−	3,5-Octadien-2-one	1063	1058	1522	1513	MS, RI	−	38284-27-4	−	−	−
32	260	5-Ethyldihydro-2(3H)-furanone	1057	1039	1694	1701	MS, RI, O, S	Sweet, coconut	695-06-7	81	−	81
33	9.7	Dihydro-5-propyl-2(3H)-furanone	1159	−	1787	1799	MS, RI, O, S	Nutty, sweet	105-21-5	1	−	−
34	9.7	Dihydro-5-pentyl-2(3H)-furanone	1363	1349	2024	2032	MS, RI, O, S	Buttery, creamy	104-61-0	27	27	9
35	12	5-Butyldihydro-2(3H)-furanone	1261	1247	1910	1921	MS, RI, O, S	Sweet, coconut	104-50-7		1	27
		Ester										
36	5	Methyl isovalerate	764	776	1018	−	MS, RI	−	556-24-1	−	−	−
37	70	Hexanoic acid, methyl ester	907	907	1184	−	MS, RI	−	106-70-7	−	−	−
38	5	Hexanoic acid, ethyl ester	1000	988	1233	1226	MS, RI, O, S	Fruity, sweet	123-66-0	3	−	1
39	200	Octanoic acid, methyl ester	1108	1111	1385	−	MS, RI	−	111-11-5	−	−	−
40	19	Octanoic acid, ethyl ester	1196	−	1435	1429	MS, RI, O, S	Waxy, fruity	106-32-1	9	−	−
41	−	Nonanoic acid, methyl ester	1208	1208	1491	−	MS, RI	−	1731-84-6	−	−	−
42	8	Decanoic acid, methyl ester	1308	1305	1593	−	MS, RI	−	110-42-9	−	−	−
43	5	Decanoic acid, ethyl ester	1379	1377	1638	−	MS, RI	−	110-38-3	−	−	−
44	−	Valeric acid, 4-pentadecyl ester	−	−	−	1505	MS	−	959021-71-7	−	−	−
45	−	n-Caproic acid vinyl ester	−	−	−	1665	MS	−	3050-69-9	−	−	−
46	781	(E)-hexanoic acid, 2-hexenyl ester	1391	−	1662	1663	MS, RI, O	Natural, green	53398-86-0	−	1	−
47	−	Hexanoic acid, pentyl ester	1270	1271	1501	1503	MS, RI	−	540-07-8	−	−	−
48	−	Isoamyl lactate	1047	−	1580	1573	MS, RI, O	Fruity, nutty	19329-89-6	−	−	1
		Aromatic compounds										
49	1000	p-Xylene	860	845	1138	−	MS, RI	−	106-42-3	−	−	−
50	350	Benzaldehyde	962	−	1520	1518	MS, RI, O	Almond, bitter	100-52-7	9	1	−
51	5000	Phenol	980	−	2000	2014	MS, RI, O	Plastic, rubber	108-95-2	1	1	3
52	31	3-Methyl-phenol	1075	−	2091	2102	MS, RI, O	Medicinal, woody	108-39-4	3	−	−
53	1000	Butylated hydroxytoluene	1513	−	1909	1925	MS, RI, O	Mild, camphor	128-37-0	−	9	−
54	30	p-Cresol	1077	−	2080	2092	MS, RI, O	Animal, mimosa	106-44-5	−	−	9
		Hydrocarbons										
55	200	Limonene	1023	1011	1200	−	MS, RI	−	138-86-3	−	−	−
56	10,000	Dodecane	1200	1197	1200	−	MS, RI, S	−	112-40-3	−	−	−
57	−	Tridecane	1300	1289	1300	−	MS, RI, S	−	629-50-5	−	−	−
58	−	2,6,10-Trimethyl-dodecane	1366	−	1354	1352	MS, RI	−	3891-98-3	−	−	−
59	−	3-Methyl-tridecane	1371	−	1366	1359	MS, RI	−	6418-41-3	−	−	−
60	1000	Tetradecane	1400	−	1400	1395	MS, RI, S	−	629-59-4	−	−	−
61	−	Pentadecane	1500	1498	1500	1494	MS, RI, S	−	629-62-9	−	−	−
62	−	Hexadecane	1600	1594	1600	1597	MS, RI, S	−	544-76-3	−	−	−
		Acids										
63	99,000	Acetic acid	610	−	1449	1451	MS, RI, O	Sharp	64-19-7	3	−	−
64	2400	Butanoic acid	805	−	1625	1633	MS, RI, O, S	Cheese, sharp	107-92-6	9	3	3
65	15.9	3-Methyl-butanoic acid	863	−	1666	1675	MS, RI, O, S	Feet, cheese	503-74-2	243	81	81
66	1207	Pentanoic acid	904	−	1733	1744	MS, RI, O, S	Rancid, putrid	109-52-4	27	1	−
67	2517.6	Hexanoic acid	990	1000	1846	1852	MS, RI, O, S	Sour, cheese	142-62-1	27	3	27
68	640	Heptanoic acid	1078	−	1950	1961	MS, RI, O, S	Sweat, sour	111-14-8	1	27	27
69	3000	Octanoic acid	1180	1183	2060	2069	MS, RI, O, S	Fatty, waxy	124-07-2	1	3	−
70	4600	Nonanoic acid	1273	−	2171	2176	MS, RI, O, S	Dirty, cheese	112-05-0	3	1	1
71	130	n-Decanoic acid	1373	1365	2276	2282	MS, RI, O	Unpleasant, fatty	334-48-5	1	3	3
72	−	(E)-2-octenoic acid	1227	−	2182	2194	MS, RI	−	1871-67-6	−	−	−
		Others										
73	−	Glycerin	−	−	2303	2305	MS, RI	−	56-81-5	−	−	−
74	5.8	2-Pentyl-furan	993	979	1231	1225	MS, RI, O	Fruity, beany	3777-69-3	−	1	−
75	−	Unknown	−	−	−	1280	O	Mushroom	−	27	9	27
76	−	Unknown	−	−	−	1568	O	Rice	−	−	−	1

RI: retention indices; MS: mass spectrometry data; O: sniff; FD factors: flavor dilution factors; S: standard; “−”: not detected.

**Table 3:** The concentrations and OAVs of volatile compounds detected via GC–O–MS.

No.	Threshold (µg/kg)	Compounds	Concentration (µg/kg)			OAV		
			XW	MN	ZZ	XW	MN	ZZ
		Aldehydes						
1	4	Hexanal	868.55 ± 41.46 ^b^	628.88 ± 28.47 ^a^	1123.21 ± 66.27 ^c^	217.14 ± 10.37 ^b^	157.22 ± 7.12 ^a^	280.8 ± 16.57 ^c^
2	0.7	Octanal	65.21 ± 4.34 ^a^	199.05 ± 4.81 ^c^	79.25 ± 9.83 ^b^	93.16 ± 6.2 ^a^	284.36 ± 6.87 ^c^	113.22 ± 14.04 ^b^
3	-	(Z)-2-heptenal	21.77 ± 1.57 ^a^	140.53 ± 3.4 ^b^	146.91 ± 9.78 ^b^	-	-	-
4	8	Nonanal	218.24 ± 15.48 ^b^	1253.94 ± 27.8 ^c^	164.1 ± 11.92 ^a^	27.28 ± 1.94 ^b^	156.74 ± 3.47 ^c^	20.51 ± 1.49 ^a^
5	4	(E)-2-octenal	44.47 ± 4.29 ^a^	58.22 ± 2.9 ^b^	50.64 ± 7.49 ^b^	11.12 ± 1.07 ^a^	14.55 ± 0.73 ^b^	12.66 ± 1.87 ^b^
6	3	Decanal	17.94 ± 0.75 ^a^	53.62 ± 4.52 ^b^	-	5.98 ± 0.25 ^a^	17.87 ± 1.51 ^b^	-
7	0.25	(E)-2-nonenal	10.39 ± 0.56 ^b^	5.66 ± 0.89 ^a^	11.35 ± 1.6 ^b^	41.55 ± 2.25 ^b^	22.65 ± 3.55 ^a^	45.38 ± 6.41 ^b^
8	1	(E,E)-2,4-nonadienal	0.7 ± 0.2	-	-	0.7 ± 0.2	-	-
9	0.2	(E,E)-2,4-decadienal	16.45 ± 0.93 ^b^	18.84 ± 0.12 ^c^	7.84 ± 0.99 ^a^	82.25 ± 4.67 ^b^	94.18 ± 0.62 ^c^	39.2 ± 4.95 ^a^
10	3	Heptanal	-	423.63 ± 11.32 ^b^	146.91 ± 9.78 ^a^	-	141.21 ± 3.77 ^b^	48.97 ± 3.26 ^a^
11	0.3	(E)-2-decenal	-	23.48 ± 2.79 ^a^	31.74 ± 2.63 ^b^	-	78.26 ± 9.3 ^a^	105.8 ± 8.77 ^b^
12	1.4	2-Undecenal	-	7.86 ± 0.97 ^a^	12.54 ± 1.19 ^b^	-	5.61 ± 0.7 ^a^	8.96 ± 0.85 ^b^
13	0.04	(E,Z)-2,4-decadienal	-	4.85 ± 0.45	-	-	121.28 ± 11.32	-
		Total	1263.73 ± 14.86 ^a^	2818.54 ± 53.99 ^c^	1774.5 ± 42.45 ^b^	576.14 ± 13.18 ^a^	950.61 ± 22.68 ^c^	675.51 ± 2.32 ^b^
		Alcohols						
14	150.2	1-Pentanol	88.36 ± 9.83 ^c^	27.48 ± 3.58 ^a^	53.34 ± 5.24 ^b^	0.59 ± 0.07 ^c^	0.18 ± 0.02 ^a^	0.36 ± 0.03 ^b^
15	1	1-Octen-3-ol	129.7 ± 5.96 ^c^	72.55 ± 1.98 ^a^	101.94 ± 3.28 ^b^	129.7 ± 5.96 ^c^	72.55 ± 1.98 ^a^	101.94 ± 3.28 ^b^
16	25,482	2-Ethyl-1-hexanol	121.04 ± 19.38	-	-	-	-	-
17	-	2,3-Butanediol	223.45 ± 0.79 ^b^	332.41 ± 2.89 ^c^	149.1 ± 33.13 ^a^	-	-	-
18	110	1-Octanol	16.89 ± 2.33 ^a^	24.76 ± 2.14 ^b^	29.74 ± 1.88 v	0.15 ± 0.02 ^a^	0.23 ± 0.02 v	0.27 ± 0.02v
19	20	(E)-2-octen-1-ol	8.6 ± 1.19 v	3.36 ± 0.39 ^a^	10.14 ± 0.98 ^c^	0.43 ± 0.06 ^b^	0.17 ± 0.02 ^a^	0.51 ± 0.05 ^c^
20	-	Isopinocarveol	9.16 ± 2.71	-	-	-	-	-
21	5.6	1-Hexanol	55.39 ± 1.27 ^b^	23.86 ± 2.38 ^a^	-	9.89 ± 0.23 ^b^	4.26 ± 0.42 ^a^	-
22	3	1-Heptanol	-	89.62 ± 1.2	-	-	29.87 ± 0.4	-
23	140	Phenylethyl alcohol	-	3.71 ± 0.96	-	-	0.03 ± 0.01	-
		Total	652.59 ± 26.12 ^c^	577.76 ± 2.6 ^b^	344.26 ± 34.04 ^a^	140.91 ± 5.65 ^b^	128.15 ± 1.86 ^a^	103.07 ± 3.31 ^c^
		Ketones						
24	40	5-Nonanone	80.76 ± 4.27 ^b^	16.51 ± 1.05 ^a^	-	2.02 ± 0.11 ^b^	0.41 ± 0.03 ^a^	-
25	110	2,3-Octanedione	92.27 ± 7.8 ^c^	28.13 ± 2.25 ^a^	51.82 ± 3.95 ^b^	0.84 ± 0.07 ^c^	0.26 ± 0.02 ^a^	0.47 ± 0.04 ^b^
26	100	(E,E)-3,5-octadien-2-one	19.56 ± 1.68 ^a^	44.56 ± 5.39 ^b^	-	0.2 ± 0.02 ^a^	0.45 ± 0.05 ^b^	-
27	65	Acetophenone	12.88 ± 0.13	-	-	0.2 ± 0	-	-
28	14	Acetoin	-	5.32 ± 0.15 ^a^	10.32 ± 1.21 ^b^	-	0.38 ± 0.01 ^a^	0.74 ± 0.09 ^b^
29	50.2	2-Octanone	-	5.28 ± 0.11	-	-	0.11 ± 0	-
30	-	(E)-3-octen-2-one	40.76 ± 5.97 ^a^	53.21 ± 5.13 ^b^	-	-	-	-
31	-	3,5-Octadien-2-one	-	15.15 ± 0.7	-	-	-	-
32	260	5-Ethyldihydro-2(3H)-furanone	24.65 ± 0.14 ^b^	17.94 ± 1.15 ^a^	-	0.09 ± 0 ^b^	0.07 ± 0 ^a^	-
33	9.7	Dihydro-5-propyl-2(3H)-furanone	2.75 ± 0.16	-	-	0.28 ± 0.02	-	-
34	9.7	Dihydro-5-pentyl-2(3H)-furanone	3.18 ± 0.1 ^b^	2.11 ± 0.89 ^a^	3.82 ± 0.07 ^c^	0.33 ± 0.01 ^b^	0.22 ± 0.09 ^a^	0.39 ± 0.01 ^c^
35	12	5-Butyldihydro-2(3H)-furanone	-	8.95 ± 0.53 ^b^	2.38 ± 0.5 ^a^	-	0.75 ± 0.04 ^b^	0.2 ± 0.04 ^a^
		Total	276.82 ± 4.36 ^c^	197.15 ± 1.33 ^b^	68.35 ± 3.3 ^a^	5.56 ± 0.06 ^c^	2.75 ± 0.07 ^b^	1.8 ± 0 ^a^
		Ester						
36	5	Methyl isovalerate	-	4.38 ± 0.21	-	-	0.88 ± 0.04	-
37	70	Hexanoic acid, methyl ester	32.72 ± 0.93 ^b^	15.62 ± 3.15 ^a^	-	0.47 ± 0.01 ^b^	0.22 ± 0.05 ^a^	-
38	5	Hexanoic acid, ethyl ester	116.07 ± 10.83 ^b^	31.22 ± 3.03 ^a^	-	23.21 ± 2.17 ^b^	6.24 ± 0.61 ^a^	-
39	200	Octanoic acid, methyl ester	17.2 ± 0.05 ^b^	4.86 ± 0.52 ^a^	-	0.09 ± 0 ^b^	0.02 ± 0 ^a^	-
40	19	Octanoic acid, ethyl ester	12.52 ± 0.42	-	-	0.66 ± 0.02	-	-
41	-	Nonanoic acid, methyl ester	-	0.38 ± 0.01	-	-	-	-
42	8	Decanoic acid, methyl ester	-	0.45 ± 0.02	-	-	0.06 ± 0	-
43	5	Decanoic acid, ethyl ester	-	1.02 ± 0.01	-	-	0.2 ± 0	-
44	-	Valeric acid, 4-pentadecyl ester	98.9 ± 12.3	-	-	-	-	-
45	-	n-Caproic acid vinyl ester	81 ± 5.47 ^b^	62.29 ± 4.85 ^a^	-	-	-	-
46	781	(E)-hexanoic acid, 2-hexenyl ester	-	-	15.8 ± 1.53	-	-	0.02 ± 0
47	-	Hexanoic acid, pentyl ester	-	4.8 ± 0.52	-	-	-	-
48	-	Isoamyl lactate	-	31.1 ± 4.07	-	-	-	-
		Total	358.4 ± 29.91 ^c^	156.11 ± 1.74 ^b^	15.8 ± 1.53 ^a^	24.43 ± 2.2 ^c^	7.63 ± 0.6 ^b^	0.02 ± 0 ^a^
		Aromatic compounds						
49	1000	p-Xylene	11.3 ± 0.75	-	-	0.01 ± 0	-	-
50	350	Benzaldehyde	82.42 ± 13.27 ^b^	-	31.1 ± 2.23 ^a^	0.24 ± 0.04 ^b^	-	0.09 ± 0.01 ^a^
51	5000	Phenol	8.12 ± 1.11 ^b^	13.48 ± 0.09 ^c^	6.64 ± 0.86 ^a^	-	-	-
52	31	3-Methyl-phenol	8.99 ± 1.02	-	-	0.29 ± 0.03	-	-
53	1000	Butylated hydroxytoluene	-	-	12.21 ± 1.91	-	-	0.01 ± 0
54	30	p-Cresol	-	3.86 ± 0.49	-	-	0.13 ± 0.02	-
		Total	110.83 ± 14.66 ^c^	17.34 ± 0.58 ^a^	49.94 ± 1.18 ^b^	0.54 ± 0.07 ^b^	0.13 ± 0.02 ^a^	0.1 ± 0 ^a^
		Hydrocarbons						
55	200	Limonene	7.19 ± 1.09 ^b^	0.38 ± 0.01 ^a^	-	0.04 ± 0.01 ^b^	0 ± 0 ^a^	-
56	10,000	Dodecane	-	3.37 ± 0.43	-	-	-	-
57	-	Tridecane	-	1.08 ± 0.05	-	-	-	-
58	-	2,6,10-Trimethyl-dodecane	-	-	35.66 ± 1.02	-	-	-
59	-	3-Methyl-tridecane	-	-	11.73 ± 1.46	-	-	-
60	1000	Tetradecane	-	-	112.5 ± 28.4	-	-	0.11 ± 0.03
61	-	Pentadecane	-	-	62.33 ± 10.48	-	-	-
62	-	Hexadecane	-	-	19.85 ± 1.51	-	-	-
		Total	7.19 ± 1.09 ^b^	4.84 ± 0.37 ^a^	242.07 ± 42.87 ^c^	0.04 ± 0.01 ^a^	-	0.11 ± 0.03 ^b^
		Acids						
63	99,000	Acetic acid	81.11 ± 4.98	-	-	-	-	-
64	2400	Butanoic acid	27.28 ± 0.17 ^c^	8.86 ± 0.39 ^a^	12.28 ± 0.84 ^b^	0.01 ± 0	-	0.01 ± 0
65	15.9	3-Methyl-butanoic acid	58.97 ± 0.99 ^c^	12.03 ± 0.23 ^a^	16.36 ± 0.71 ^b^	3.71 ± 0.06 ^c^	0.76 ± 0.01 ^a^	1.03 ± 0.04 ^b^
66	1207	Pentanoic acid	15.3 ± 0.71 ^c^	1.4 ± 0.06 ^a^	4.1 ± 0.23 ^b^	0.01 ± 0	-	-
67	2517.6	Hexanoic acid	234.84 ± 5.36 ^c^	180.33 ± 11.5 ^b^	44.05 ± 2.71 ^a^	0.09 ± 0 ^c^	0.07 ± 0 ^b^	0.02 ± 0 ^a^
68	640	Heptanoic acid	1.49 ± 0.6 ^a^	3.28 ± 1.68 ^b^	3.79 ± 0.73 ^b^	-	0.01 ± 0	0.01 ± 0
69	3000	Octanoic acid	60.03 ± 9.37 ^c^	25.63 ± 1.63 ^b^	13.54 ± 0.28 ^a^	0.02 ± 0 ^b^	0.01 ± 0 ^a^	-
70	4600	Nonanoic acid	27.1 ± 0.2 ^c^	10.31 ± 0.81 ^b^	4.9 ± 0.08 ^a^	0.01 ± 0	-	-
71	130	n-Decanoic acid	12.27 ± 0.59 ^a^	22.79 ± 2.41 ^c^	19.27 ± 1.31 ^b^	0.09 ± 0 ^a^	0.18 ± 0.02 ^c^	0.15 ± 0.01 ^b^
72	-	(E)-2-octenoic acid	-	2.43 ± 0.2	-	-	-	-
		Total	518.38 ± 16.47 ^c^	267.06 ± 7.57 ^b^	118.29 ± 0.28 ^a^	4.53 ± 0.2 ^b^	4.51 ± 0.41 ^b^	1.21 ± 0.05 ^a^
		Others						
73	-	Glycerin	105.37 ± 22.25 ^c^	15.31 ± 0.34 ^b^	10.71 ± 0.19 ^a^	-	-	-
74	5.8	2-Pentyl-furan	-	3.58 ± 2 ^a^	8.76 ± 0.76 ^b^	-	0.62 ± 0.34 ^a^	1.51 ± 0.13 ^b^
		Total	105.37 ± 22.25 ^b^	18.89 ± 1.65 ^a^	19.46 ± 0.57 ^a^	-	2.34 ± 0.34 ^b^	1.51 ± 0.13 ^a^

OAV: odor activity value. Different letters in the same row indicate significant differences (*p* < 0.05). “-”: no threshold can be found or compounds were not detected.

**Table 4:** Authentic standards, quantitative ions, and standard curves in determination of volatile compounds in selected ion monitoring mode.

Compounds	Quantitative Ions ^a^	Standard Curves ^b^	R^2^
Hexanal	44, 56, 72, 27	y = 0.2735x + 0.1596	0.9932
Nonanal	57, 41, 43, 56	y = 0.4929x − 0.1045	0.9979
Heptanal	44, 27, 55, 70	y = 0.2328x − 0.2125	0.9968
Decanal	56, 55, 41, 43	y = 0.9334x − 0.2805	0.9861
Octanal	43, 44, 41, 56	y = 0.1527x − 0.7610	0.9968
(E)-2-Nonenal	41, 43, 29, 55	y = 0.3585x + 0.1831	0.9978
(E,E)-2,4-Decadienal	81, 41, 29, 39	y = 0.3159x + 0.1903	0.9956
2-Undecenal	70, 41, 57, 43	y = 0.3336x + 0.1133	0.9975
1-Octen-3-ol	57, 43, 72, 29	y = 0.3077x + 0.2273	0.9974
1-Hexanol	56, 43, 41, 39	y = 0.3201x − 0.2449	0.9969
1-Octanol	56, 55, 41, 43	y = 0.6931x − 0.4813	0.9901
2,3-Butanediol	45, 43, 27, 57	y = 0.4102x − 0.1892	0.9924
2,3-Octanedione	43, 30, 41, 27	y = 0.1776x − 0.3727	0.9971
Butyldihydro-2(3H)-furanone	85, 29, 56, 41	y = 0.5138x − 0.2752	0.9858
Dihydro-5-pentyl-2(3H)-furanone	85, 29, 41, 43	y = 0.4636x + 0.1317	0.9988
5-Ethyldihydro-2(3H)-furanone	85, 29, 56, 57	y = 0.2432x − 0.1592	0.9986
3-Methyl-butanoic acid	60, 43, 41, 45	y = 0.1087x − 0.3425	0.9911
Pentanoic acid	60, 73, 41, 45	y = 0.8277x − 0.2338	0.9878
Hexanoic acid	60, 73, 41, 43	y = 0.6026x − 0.8420	0.9759
Heptanoic acid	60, 73, 41, 87	y = 0.2074x + 0.6202	0.9972

^a^: Monitored ions used for quantitation. ^b^: Variables: x and y represent the concentration ratio (the concentration of the standard of volatile compounds/internal standard) and the peak area ratio (the peak area of standard/internal standard).

**Table 5:** The results of the triangle test via omission experiments.

No.	Compounds ^a^	Correct Number ^b^	Significance ^c^
Yunnan bacon			
1	Hexanal	12/12	**
2	(E)-2-Nonenal	5/12	–
3	(E,E)-2,4-Decadienal	8/12	*
4	1-Octen-3-ol	12/12	**
5	2,3-Butanediol	6/12	–
6	1-Hexanol	8/12	*
7	2,3-Octanedione	9/12	**
8	5-Ethyldihydro-2(3H)-furanone	3/12	–
9	Dihydro-5-pentyl-2(3H)-furanone	8/12	*
10	3-Methyl-butanoic acid	10/12	**
11	Pentanoic acid	4/12	–
12	Hexanoic acid	9/12	**
Chongqing bacon			
1	Hexanal	12/12	**
2	(E)-2-Nonenal	9/12	**
3	(E,E)-2,4-Decadienal	8/12	*
4	2-Undecenal	9/12	**
5	1-Octen-3-ol	12/12	**
6	2,3-Octanedione	10/12	**
7	Dihydro-5-pentyl-2(3H)-furanone	8/12	*
8	3-Methyl-butanoic acid	8/12	*
9	Heptanoic acid	6/12	–
Sichuan bacon			
1	Hexanal	12/12	**
2	Octanal	11/12	**
3	Nonanal	7/12	–
4	Decanal	6/12	–
5	(E,E)-2,4-Decadienal	8/12	*
6	Heptanal	5/12	–
7	2-Undecenal	8/12	*
8	1-Octen-3-ol	12/12	**
9	2,3-Butanediol	4/12	–
10	1-Octanol	10/12	**
11	5-Ethyldihydro-2(3H)-furanone	3/12	–
12	5-Butyldihydro-2(3H)-furanone	9/12	**
13	3-Methyl-butanoic acid	10/12	**
14	Hexanoic acid	9/12	**
15	Heptanoic acid	6/12	–

^a^ The aroma compounds with FD factors greater than 27. ^b^ The number of correct judgements from 12 panelists. ^c^ ** highly significant (*p* < 0.01); * significant (*p* < 0.05); – no significant difference.

## Data Availability

The original contributions presented in the study are included in the article/Supplementary Materials, further inquiries can be directed to the corresponding author.

## Supplementary Materials

The following supporting information can be downloaded at: https://www.mdpi.com/article/10.3390/foods13081260/s1, Table S1: Volatile compounds in non-smoked bacon detected by GC × GC-TOFMS.
