# Supplementary material for: The Characterization of the Key Aroma Compounds in Non-Smoked Bacon by Instrumental and Sensory Methods
Source: Foods. 2024 Apr 19;13(8):1260. doi: 10.3390/foods13081260 (PMC11049224; doi:10.3390/foods13081260)
Supplement: Supplementary file 1 [file foods-13-01260-s001.zip › foods-2957993-supplementary.pdf]

Table S1. Volatile compounds in non-smoked bacon detected by GC × GC-TOFMS.

NO.	CAS	Compounds	RI	1st Dt (min)	2nd Dt (s)	Concentration (μg/kg)		
						XW	ZZ	MN
		Hydrocarbons						
1	109-66-0	Pentane	500	4.10	1.60	560.84±43.79^{b}	700.73±43.26^{c}	214.48±12.86^{a}
2	592-76-7	1-Heptene	746	4.90	1.92	13.65±0.52^{a}	72.78±5.12^{b}	-
3	111-65-9	Octane	791	5.40	2.30	377.27±33.67^{a}	886.27±32.47^{b}	-
4	7642-15-1	(Z)-4-Octene	846	6.20	2.36	10.54±0.46	-	-
5	13389-42-9	(E)-2-Octene	860	6.40	2.42	5.89±0.55	-	-
6	111-67-1	2-Octene	846	6.20	2.38	-	55.49±4.66^{b}	32.70±2.16^{a}
7	14850-22-7	(Z)-3-Octene	860	6.40	2.42	-	28.14±2.36	-
8	111-84-2	Nonane	893	6.90	2.86	5.81±0.43^{a}	10.32±0.39^{b}	18.09±1.64^{c}
9	1465084	3-Methyl-nonane	962	8.50	3.32	14.80±1.56	-	-
10	124-18-5	Decane	1000	9.40	3.50	84.53±6.96^{b}	39.04±2.68^{a}	572.54±48.18^{c}
11	872-05-9	1-Decene	1039	10.70	3.34	39.09±3.52	-	-
12	56700-77-7	(E)-1,3-nonadiene	1051	11.10	2.94	4.68±0.42	-	-
13	1120-21-4	Undecane	1088	12.30	4.18	66.11±5.02^{a}	69.99±7.52^{a}	69.60±6.96^{a}
14	112-40-3	Dodecane	1186	16.30	4.58	141.56±8.95^{a}	126.11±10.99^{a}	233.93±10.38^{b}
15	103-65-1	Propyl-benzene	1199	16.90	2.92	238.26±16.92^{b}	163.67±10.21^{a}	163.76±17.81^{a}
16	300-57-2	2-Propenyl-benzene	1257	19.40	2.66	57.37±3.26^{c}	36.23±2.39^{b}	22.22±2.09^{a}
17	629-50-5	Tridecane	1293	20.90	4.62	76.61±6.81	366.68±28.3	54.84±4.15
18	3891-98-3	2,6,10-Trimethyl-dodecane	1351	23.40	4.82	-	270.57±23.96	-
19	6418-41-3	3-Methyl-tridecane	1360	23.80	4.68	8.95±0.96^{a}	-	7.82±0.62^{a}
20	629-59-4	Tetradecane	1393	25.20	4.66	27.24±2.72^{a}	1201.47±98.93^{b}	31.91±2.47^{a}

21	41446-78-0	(E)-4-tetradecene	1439	27.10	4.68	-	31.37±2.6	-
22	4861-58-9	2-Pentyl-thiophene	1453	27.70	3.06	2.48±0.25	-	-
23	544-76-3	Hexadecane	1597	33.50	4.64	-	213.86±14.82^{b}	2.97±0.14^{a}
		SubTotal				1735.66±136.77^{b}	4272.73±290.66^{c}	1424.87±109.45^{a}
	Aldehydes							
24	123-38-6	Propanal	781	5.30	1.72	897.87±89.94	-	-
25	78-84-2	2-Methyl-propanal	806	5.60	1.80	10.51±0.82	-	-
26	123-72-8	Butanal	866	6.50	1.86	50.09±2.16^{b}	67.27±5.32^{c}	31.56±2.35^{a}
27	96-17-3	2-Methyl-butanal	908	7.20	2.00	4.94±0.27^{a}	4.1±0.22^{a}	60.82±4.76^{b}
28	590-86-3	3-Methyl-butanal	913	7.30	3.32	227.9±15.07^{b}	276.51±22.71^{c}	181.92±11.37^{a}
29	110-62-3	Pentanal	974	8.80	2.14	1359.41±91.43^{b}	3756.3±247.58^{c}	935.11±78.94^{a}
30	15798-64-8	(Z)-2-butenal	1035	10.60	1.94	15.64±1.47^{a}	20.72±1.80^{b}	-
31	66-25-1	Hexanal	1081	12.10	2.62	2192.00±195.71^{b}	2774.38±186.79^{c}	756.13±75.48^{a}
32	497-03-0	(E)-2-methyl-2-butenal	1093	12.50	2.16	-	49.38±4.52	-
33	1115-11-3	2-Methyl-2-butenal	1093	12.50	2.16	15.99±1.05	-	-
34	1576-87-0	(E)-2-pentenal	1128	13.90	2.14	28.25±2.59^{b}	19.29±0.54^{a}	16.56±1.00^{a}
35	623-36-9	2-Methyl-2-pentenal	1154	15.00	2.36	1.17±0.09	-	-
36	111-71-7	Heptanal	1178	16.00	2.64	899.25±74.20^{a}	1042.42±102.46^{a}	883.00±43.92^{a}
37	6728-26-3	(E)-2-hexenal	1213	17.50	2.30	120.01±11.05^{c}	72.36±6.14^{b}	19.82±1.42^{a}
38	6728-31-0	(Z)-4-heptenal	1234	18.40	2.42	9.48±0.77^{c}	8.07±0.72^{b}	4.33±0.37^{a}
39	124-13-0	Octanal	1285	20.60	2.68	728.11±67.84^{b}	416.14±14.95^{a}	337.25±24.96^{a}
40	18829-55-5	(E)-2-heptenal	1320	22.10	2.42	308.96±23.84^{c}	261.37±13.44^{b}	204.22±21.13^{a}
41	645-62-5	2-Ethyl-2-hexenal	1329	22.50	2.64	3.05±0.29^{a}	13.26±0.66^{b}	-
42	124-19-6	Nonanal	1390	25.10	2.82	1644.88±131.47^{c}	1132.33±77.96^{b}	207.42±16.61^{a}
43	142-83-6	(E,E)-2,4-hexadienal	1397	25.40	2.10	3.79±0.31^{b}	2.65±0.25^{a}	-

44	2548-87-0	(E)-2-octenal	1426	26.60	2.48	236.69±20.39[c]	160.46±23.12[b]	83.72±7.51[a]
45	3268-49-3	Methional	1448	27.50	2.12	36.61±2.39[a]	–	34.37±1.80[a]
46	98-01-1	Furfural	1455	27.80	1.88	2.76±0.22	–	–
47	4313-03-5	(E,E)-2,4-heptadienal	1489	29.20	2.18	38.12±3.15[c]	25.32±1.76[b]	18.25±1.07[a]
48	112-31-2	Decanal	1494	29.40	2.82	13.21±1.60[a]	18.01±1.79[b]	28.78±1.45[c]
49	557-48-2	(E,Z)-2,6-nonadienal	1582	32.90	2.42	4.16±0.40[c]	2.00±0.08[b]	0.72±0.06[a]
50	3913-81-3	(E)-2-decenal	1642	35.20	2.56	–	–	12.14±1.01
51	90-02-8	2-Hydroxy-benzaldehyde	1672	36.30	2.08	2.15±0.24	–	–
52	5392-40-5	Neral	1680	36.60	2.52	1.89±0.22[b]	1.26±0.10[a]	–
53	6750-03-4	2,4-Nonadienal	1699	37.30	2.32	61.97±2.63	–	–
54	5910-87-2	(E,E)-2,4-nonadienal	1699	37.30	2.30	–	26.77±1.57[b]	17.31±1.13[a]
55	2463-77-6	2-Undecenal	1750	39.20	2.60	11.19±0.76[c]	8.64±0.29[b]	3.69±0.33[a]
56	25152-83-4	(E,Z)-2,4-decadienal	1761	39.60	2.38	–	–	4.84±0.42
57	2363-88-4	2,4-Decadienal	1809	41.30	2.28	34.74±2.99	–	–
58	25152-84-5	(E,E)-2,4-decadienal	1809	41.30	2.28	–	19.60±1.22[b]	16.63±0.99[a]
		SubTotal				8964.78±797.66[b]	10178.60±379.39[c]	3858.59±306.47[a]
		Alcohols						
59	75-65-0	2-Methyl-2-propanol	899	7.00	1.70	–	5.82±0.28	–
60	64-17-5	Ethanol	928	7.70	1.76	2563.92±164.76[a]	–	2330.89±296.62[a]
61	78-92-2	2-Butanol	1023	10.20	1.72	3.83±0.32	–	–
62	71-23-8	1-Propanol	1035	10.60	1.70	20.66±1.63[b]	13.62±1.05[a]	19.31±1.37[b]
63	78-83-1	2-Methyl-1-propanol	1104	12.90	1.82	–	–	11.99±0.95
64	584-02-1	3-Pentanol	1118	13.50	1.84	10.84±0.97[a]	12.93±1.16[b]	–
65	107-18-6	2-Propen-1-ol	1123	13.70	1.64	2.49±0.15	–	–

66	107-98-2	1-Methoxy-2-propanol	1137	14.30	1.82	-	-	102.40±8.69
67	71-36-3	1-Butanol	1149	14.80	1.76	-	123.68±7.68	-
68	616-25-1	1-Penten-3-ol	1161	15.30	1.78	650.19±50.19^{c}	429.14±24.97^{b}	241±19.67^{a}
69	623-37-0	3-Hexanol	1197	16.80	1.94	-	0.98±0.07^{b}	0.69±0.08^{a}
70	123-51-3	3-Methyl-1-butanol	1208	17.30	1.82	73.93±7.76^{c}	12.66±1.09^{a}	53±1.44^{b}
71	71-41-0	1-Pentanol	1250	19.10	1.92	736.61±74.92^{c}	591.59±47.73^{b}	300.81±15.77^{a}
72	589-82-2	3-Heptanol	1294	21.00	2.02	2.75±0.13^{a}	6.09±0.59^{b}	2.24±0.15^{a}
73	1576-95-0	(Z)-2-penten-1-ol	1317	22.00	1.78	295.34±19.37^{c}	212.99±15.8^{b}	55.51±4.18^{a}
74	4938-52-7	1-Hepten-3-ol	1348	23.30	1.92	5.78±0.46^{b}	5.84±0.56^{b}	3.08±0.18^{a}
75	111-27-3	1-Hexanol	1350	23.40	1.92	652.05±73.3^{b}	318.06±16.42^{a}	403.63±38.32^{a}
76	544-12-7	3-Hexen-1-ol	1380	24.70	1.88	1.90±0.17	-	-
77	589-98-0	3-Octanol	1392	25.20	2.10	1.96±0.14^{a}	2.65±0.27^{b}	3.58±0.26^{c}
78	3391-86-4	1-Octen-3-ol	1448	27.50	2.02	2255.31±86.85^{c}	430.83±28.7^{a}	1252.59±118.65^{b}
79	111-70-6	1-Heptanol	1453	27.70	1.98	232.86±20.47^{b}	273.43±18.16^{c}	73.95±3.73^{a}
80	104-76-7	2-Ethyl-1-hexanol	1487	29.10	2.02	38.85±1.95^{b}	789.45±74.19^{c}	12.18±1.26^{a}
81	33467-76-4	(E)-2-hepten-1-ol	1509	30.00	1.90	8.46±0.41^{b}	0.44±0.05^{a}	-
82	513-85-9	2,3-Butanediol	1536	31.10	1.66	-	-	446.07±19.18
83	111-87-5	1-Octanol	1556	31.90	2.02	132.41±10.59^{b}	150.47±5.44^{b}	104.67±10.44^{a}
84	18409-17-1	(E)-2-octen-1-ol	1612	34.10	1.96	117.26±8.70^{b}	155.79±11.06^{c}	25.37±1.28^{a}
85	98-00-0	2-Furanmethanol	1653	35.60	1.68	0.46±0.02^{a}	3.45±0.20^{b}	-
86	143-08-8	1-Nonanol	1658	35.80	2.10	0.50±0.04^{a}	-	1.55±0.13^{b}
87	505-10-2	3-(Methylthio)-1-propanol	1715	37.90	1.82	-	-	2.08±0.13
88	100-51-6	Benzyl alcohol	1874	43.10	1.66	24.05±2.86^{b}	7.03±0.6^{a}	5.2±0.44^{a}
89	60-12-8	Phenylethyl alcohol	1909	44.00	1.68	9.12±0.46^{b}	2.37±0.17^{a}	26.92±1.94^{c}

		SubTotal				7841.54±770.42[c]	3549.26±300.01[a]	5478.71±387.92[b]
	Ketones							
90	78-93-3	2-Butanone	892	6.90	1.86	268.6±23.89	-	-
91	1002-33-1	1,3-Octadiene	949	8.20	2.50	22.02±1.54[b]	26.95±2.18[c]	15.48±1.52[a]
92	431-03-8	2,3-Butanedione	966	8.60	1.80	443.28±31.18[a]	-	1358.91±75.03[c]
93	589-38-8	3-Hexanone	1048	11.00	2.34	1±0.1	-	-
94	600-14-6	2,3-Pentanedione	1054	11.20	1.98	455.91±52.15[a]	395.78±41.36[a]	484.65±41.35[a]
95	625-33-2	3-Penten-2-one	1125	13.80	2.10	-	0.95±0.09	-
96	106-35-4	3-Heptanone	1149	14.80	2.58	9.97±0.77[b]	17.87±1.46[c]	5.75±0.39[a]
97	110-43-0	2-Heptanone	1178	16.00	2.56	603.53±54.35[b]	508.25±30.12[a]	685.39±34.85[b]
98	589-63-9	4-Octanone	1220	17.80	2.80	0.79±0.07[a]	1.79±0.17[b]	0.76±0.05[a]
99	928-68-7	6-Methyl-2-heptanone	1232	18.30	2.60	20.10±1.56[a]	24.3±2.40[b]	-
100	106-68-3	3-Octanone	1250	19.10	2.78	35.26±1.66[b]	-	23.26±1.61[a]
101	111-13-7	2-Octanone	1281	20.40	2.64	57.96±5.72[c]	38.45±3.37[b]	21.48±2.01[a]
102	513-86-0	Acetoin	1283	20.50	1.78	379.27±27.03[a]	588.16±36.53[b]	1003.81±90.27[c]
103	108-94-1	Cyclohexanone	1290	20.80	2.52	0.57±0.05	-	-
104	116-09-6	1-Hydroxy-2-propanone	1294	21.00	1.76	-	-	38.51±2.73
105	4312-99-6	1-Octen-3-one	1297	21.10	2.54	80.39±4.57[b]	44.89±3.72[a]	37.82±2.88[a]
106	585-25-1	2,3-Octanedione	1327	22.40	2.36	8.60±0.84[a]	225.17±12.54[b]	959.45±94.7[c]
107	110-93-0	6-Methyl-5-hepten-2-one	1332	22.60	2.50	95.14±5.42[b]	17.27±1.33[a]	-
108	821-55-6	2-Nonanone	1385	24.90	2.72	26.58±2.73[b]	15.28±1.34[a]	85.64±7.82[c]
109	18402-82-9	(E)-3-octen-2-one	1404	25.70	2.48	139.09±11.80	-	-
110	1669-44-9	3-Octen-2-one	1404	25.70	2.46	-	90.79±9.19[a]	253.65±24.35[b]

111	693-54-9	2-Decanone	1492	29.30	2.78	-	8.29±0.79^{a}	7.62±0.36^{a}
112	14309-57-0	3-Nonen-2-one	1509	30.00	2.54	2.19±0.14^{b}	1.75±0.20^{a}	3.06±0.23^{c}
113	38284-27-4	3,5-Octadien-2-one	1516	30.30	2.34	19.21±1.68	-	-
114	30086-02-3	(E,E)-3,5-octadien-2-one	1566	32.30	2.26	22.42±1.42^{a}	22.62±1.77^{a}	51.26±3.69^{b}
115	108-29-2	Dihydro-5-methyl-2(3H)-furanone	1604	33.80	2.04	8.93±0.73	-	-
116	96-48-0	Butyrolactone	1623	34.50	2.00	175.17±11.61^{a}	-	221.09±22.79^{b}
117	98-86-2	Acetophenone	1645	35.30	2.22	10.48±0.6^{b}	15.98±1.34^{c}	8.04±0.61^{a}
118	695-06-7	5-Ethyldihydro-2(3H)-furanone	1699	37.30	2.14	197.52±11.86^{c}	71.2±7.34^{a}	173.34±11.48^{b}
119	105-21-5	Dihydro-5-propyl-2(3H)-furanone	1799	41.00	2.16	22.43±1.37^{b}	-	18.05±1.55^{a}
120	104-50-7	5-Butyldihydro-2(3H)-furanone	1919	44.20	1.88	44.99±3.23^{b}	38.2±1.50^{a}	46.28±1.86^{b}
121	104-61-0	Dihydro-5-pentyl-2(3H)-furanone	2032	46.30	1.78	18.84±1.53^{a}	19.4±1.92^{a}	44.97±4.08^{b}
		SubTotal				3170.27±307.85^{b}	2173.32±150.79^{a}	5548.27±398.88^{c}
	Ester							
122	79-20-9	Acetic acid, methyl ester	819	5.80	1.72	-	-	770.1±63.54
123	141-78-6	Ethyl Acetate	879	6.70	1.86	-	47.47±3.56^{a}	455.46±38.71^{b}
124	554-12-1	Methyl propionate	899	7.00	1.92	-	-	46.18±3.03
125	623-42-7	Butanoic acid, methyl ester	978	8.90	2.18	33.68±3.18^{b}	-	23.25±1.04^{a}
126	556-24-1	Methyl isovalerate	1014	9.90	2.30	14.84±1.28^{a}	-	81.9±5.39^{b}

127	105-54-4	Butanoic acid, ethyl ester	1033	10.50	2.42	48.07±3.85[c]	3.21±0.36[a]	17.12±1.66[b]
128	123-86-4	Acetic acid, butyl ester	1069	11.70	2.38	10.85±0.82[b]	9.61±0.58[a]	–
129	539-82-2	Pentanoic acid, ethyl ester	1128	13.90	2.72	15.73±1.31[b]	–	11.52±0.87[a]
130	590-01-2	Propanoic acid, butyl ester	1135	14.20	2.74	5.15±0.33[a]	6.02±0.48[b]	–
131	628-63-7	Acetic acid, pentyl ester	1166	15.50	2.62	1.82±0.14	–	–
132	106-70-7	Hexanoic acid, methyl ester	1180	16.10	2.68	589.89±18.80[b]	238.80±12.71[a]	702.58±34.62[c]
133	123-66-0	Hexanoic acid, ethyl ester	1227	18.10	2.90	236.47±15.05[c]	44.04±3.17[a]	148.82±7.3[b]
134	142-92-7	Acetic acid, hexyl ester	1267	19.80	2.74	3.66±0.31	–	–
135	540-18-1	Butanoic acid, pentyl ester	1311	21.70	3.08	2.13±0.19	–	–
136	106-30-9	Heptanoic acid, ethyl ester	1330	22.50	3.00	4.78±0.48[c]	0.89±0.08[a]	3.01±0.23[b]
137	111-11-5	Octanoic acid, methyl ester	1385	24.90	2.86	16.98±1.46[b]	–	11.75±0.81[a]
138	626-82-4	Hexanoic acid, butyl ester	1409	25.90	3.20	0.93±0.07	–	–
139	106-32-1	Octanoic acid, ethyl ester	1431	26.80	3.08	24.82±2.48[b]	–	3.56±0.32[a]

140	110-42-9	Decanoic acid, methyl ester	1592	33.30	2.98	2.14±0.21[a]	-	3.27±0.37[b]
141	110-38-3	Decanoic acid, ethyl ester	1637	35.00	3.16	4.72±0.34	-	-
142	3050-69-9	n-Caproic acid vinyl ester		35.80	1.98	-	-	186.2±13.66
		SubTotal				1016.66±82.57[b]	350.05±4.90[a]	2464.73±76.56[c]
	Aromatic compounds							
143	106-42-3	p-Xylene	1123	13.70	2.68	2108.89±147.19[c]	507.44±24.94[b]	214.72±20.05[a]
144	100-41-4	Ethylbenzene	1130	14.00	2.68	667.94±45.45[b]	1764.59±155.68[c]	351.08±18.66[a]
145	95-47-6	o-Xylene	1187	16.40	2.60	3.57±0.32[a]	7.60±0.48[b]	-
146	100-42-5	Styrene	1246	18.90	2.44	-	123.10±8.02[b]	5.57±0.19[a]
147	99-87-6	p-Cymene	1262	19.60	3.06	17.98±1.24	-	-
148	496-11-7	Indane	1364	24.00	2.94	-	-	1.17±0.12
149	100-52-7	Benzaldehyde	1516	30.30	2.16	566.38±61.84[c]	438.95±20.93[b]	175.92±12.77[a]
150	122-78-1	Benzeneacetaldehyde	1634	34.90	2.16	159.35±12.70[c]	112.73±12.71[b]	97.25±6.23[a]
151	91-20-3	Naphthalene	1737	38.70	2.50	-	6.26±0.60	-
152	275-51-4	Azulene	1737	38.70	2.50	5.11±0.36	-	-
153	108-95-2	Phenol	1994	45.70	1.48	10.15±0.58[a]	30.3±1.20[b]	48.34±3.72[c]
154	106-44-5	p-Cresol	2079	47.00	1.48	7.08±0.55[b]	2.7±0.24[a]	6.91±0.86[b]
155	120-72-9	Indole	2458	51.40	1.58	1.75±0.14[a]	6.63±0.47[b]	-
		SubTotal				3548.21±278.7[c]	3000.31±229.71[b]	900.96±35.00[a]
	Acids							
156	64-19-7	Acetic acid	1443	27.30	1.52	508.20±48.26[b]	212.20±19.48[a]	637.14±55.08[c]
157	79-09-4	Propanoic acid	1536	31.10	1.54	38.35±3.21[a]	-	55.98±3.37[b]

158	107-92-6	Butanoic acid	1623	34.50	1.58	465.95±37.93[c]	36.9±2.21[a]	123.92±12.55[b]
159	503-74-2	3-Methyl-butanoic acid	1669	36.20	1.62	145.47±10.49[b]	57.06±4.15[a]	247.47±16.72[c]
160	109-52-4	Pentanoic acid	1736	38.70	1.60	176.98±17.87[c]	39.98±4.14[a]	129.52±13.16[b]
161	142-62-1	Hexanoic acid	1845	42.30	1.62	981.35±102.55[b]	357.55±11.64[a]	1656.48±164.28[c]
162	111-14-8	Heptanoic acid	1959	45.00	1.52	45.43±3.22[b]	16.71±1.05[a]	41.78±2.54[b]
163	124-07-2	Octanoic acid	2066	46.80	1.50	139.81±16.87[c]	35.62±1.88[a]	78.72±4.80[b]
164	112-05-0	Nonanoic acid	2176	48.30	1.48	16.11±1.34[b]	8.82±0.64[a]	18.1±2.53[b]
165	334-48-5	n-Decanoic acid	2290	49.60	1.48	-	5.7±0.46[a]	16.39±0.32[b]
		SubTotal				2517.63±182.19[b]	770.55±58.62[a]	3005.49±215.8[c]
	Furan							
166	110-00-9	Furan	790	5.40	1.66	2.31±0.16[c]	1.37±0.07[b]	1.07±0.04[a]
167	930-27-8	3-Methyl-furan	859	6.40	1.80	3.66±0.23	-	-
168	534-22-5	2-Methyl-furan	859	6.40	1.80	-	3.6±0.26[b]	2.81±0.20[a]
169	3208-16-0	2-Ethyl-furan	945	8.10	2.04	41.24±3.23[c]	20.49±1.55[a]	31.43±2.26[b]
170	4229-91-8	2-Propyl-furan	1027	10.30	2.32	4.18±0.19[a]	3.85±0.37[a]	5.3±0.30[b]
171	3777-69-3	2-Pentyl-furan	1220	17.80	2.88	640.43±33.06[b]	586.18±57.44[b]	202.73±17.54[a]
172	3777-70-6	2-Hexyl-furan	1323	22.20	2.98	2.09±0.09	-	-
173	3777-71-7	2-n-Heptylfuran	1426	26.60	3.08	1.32±0.12	-	-
		SubTotal				695.23±67.15[b]	615.49±25.36[b]	243.34±14.14[a]
	Others							
174	110-86-1	Pyridine	1187	16.40	2.10	34.13±3.93[b]	44.45±3.88[c]	25.54±2.54[a]
175	470-82-6	Eucalyptol	1207	17.20	3.54	-	8.84±0.55	-
176	290-37-9	Pyrazine	1211	17.40	2.04	8.14±0.76[a]	56.54±4.23[b]	-
177	3581-87-1	2-Methyl-thiazole	1236	18.50	2.20	8.77±1.06[b]	7.5±0.62[b]	4.72±0.42[a]
178	288-47-1	Thiazole	1245	18.90	1.98	16.10±1.16[c]	6.44±0.54[b]	4.83±0.36[a]

179	109-08-0	Methyl-pyrazine	1264	19.70	2.20	–	7.93±0.82[b]	6.01±0.38[a]
180	15679-09-1	2-Ethyl-thiazole	1301	21.30	2.38	5.66±0.22	–	–
181	108-50-9	2,6-Dimethyl-pyrazine	1327	22.40	2.36	–	–	19.02±1.83
182	5910-89-4	2,3-Dimethyl-pyrazine	1346	23.20	2.34	–	–	29.09±2.68
183	3658-80-8	Dimethyl trisulfide	1374	24.40	2.68	–	–	4.11±0.40
184	14667-55-1	Trimethyl-pyrazine	1404	25.70	2.50	1.63±0.10[a]	–	111.01±10.77[b]
185	1124-11-4	Tetramethyl-pyrazine	1477	28.70	2.64	2.31±0.23	–	–
186	109-97-7	Pyrrole	1506	29.90	1.66	–	5.24±0.31[a]	23.61±1.74[b]
187	2294-76-0	2-Pentyl-pyridine	1577	32.70	2.74	–	–	2.19±0.16
188	95-16-9	Benzothiazole	1959	45.00	1.86	0.35±0.03[a]	–	0.34±0.02[a]
		SubTotal				77.08±8.60[a]	136.94±12.42[b]	230.46±13.14[c]
		Total				48433.69±3905.61[c]	35643.17±2021.5[a]	41027.38±2457.88[b]

1st Dt and 2nd Dt means 1st dimension time (min) and 2nd dimension time (s), respectively. "–": Not detected; RI: Retention indices. Different letters in the same row indicate significant differences ($P < 0.05$).
